# Two components of the *rhpPC* operon coordinately regulate the type III secretion system and bacterial fitness in *Pseudomonas savastanoi* pv. *phaseolicola*

**DOI:** 10.1371/journal.ppat.1007673

**Published:** 2019-04-18

**Authors:** Kun Li, Yanan Zhu, Wei Yan, Xin Deng, Yanmei Xiao, Liyang Song, Rongxiang Fang, Yantao Jia, Xiaoyan Tang

**Affiliations:** 1 Guangdong Key Lab of Biotechnology for Plant Development, School of Life Sciences, South China Normal University, Guangzhou, China; 2 State Key Laboratory of Plant Genomics, Institute of Microbiology, Chinese Academy of Sciences, Beijing, China; 3 National Plant Gene Research Center, Beijing, China; 4 School of Life Sciences, Capital Normal University, Beijing, China; 5 Department of Biomedical Sciences, City University of Hong Kong, Kowloon Tong, Hong Kong; 6 Department of Plant Pathology, Kansas State University, Kansas, United States of America; 7 College of Life Sciences, University of the Chinese Academy of Sciences, Beijing, China; University of California Riverside, UNITED STATES

## Abstract

Many plant bacterial pathogens including *Pseudomonas* species, utilize the type III secretion system (T3SS) to deliver effector proteins into plant cells. Genes encoding the T3SS and its effectors are repressed in nutrient-rich media but are rapidly induced after the bacteria enter a plant or are transferred into nutrient-deficient media. To understand how the T3SS genes are regulated, we screened for *P*. *savastanoi* pv. *phaseolicola* (*Psph*) mutants displaying diminished induction of *avrPto-luc*, a reporter for the T3SS genes, in *Arabidopsis*. A mutant carrying transposon insertion into a gene coding for a small functional unknown protein, designated as *rhpC*, was identified that poorly induced *avrPto-luc* in plants and in minimal medium (MM). Interestingly, *rhpC* is located immediately downstream of a putative metalloprotease gene named *rhpP*, and the two genes are organized in an operon *rhpPC*; but *rhpP* and *rhpC* displayed different RNA expression patterns in nutrient-rich King’s B medium (KB) and MM. Deletion of the whole *rhpPC* locus did not significantly affect the *avrPto-luc* induction, implying coordinated actions of *rhpP* and *rhpC* in regulating the T3SS genes. Further analysis showed that RhpC was a cytoplasmic protein that interacted with RhpP and targeted RhpP to the periplasm. In the absence of RhpC, RhpP was localized in the cytoplasm and caused a reduction of HrpL, a key regulator of the T3SS genes, and also reduced the fitness of *Psph*. Expression of RhpP alone in *E*. *coli* inhibited the bacterial growth. The detrimental effect of RhpP on the fitness of *Psph* and *E*. *coli* required metalloprotease active sites, and was repressed when RhpC was co-expressed with RhpP. The coordination between *rhpP* and *rhpC* in tuning the T3SS gene expression and cell fitness reveals a novel regulatory mechanism for bacterial pathogenesis. The function of RhpP in the periplasm remains to be studied.

## Author summary

The induction of the type III secretion system (T3SS) is of great importance to the pathogenesis of bacterial pathogens in host plants. *Pseudomonas savastanoi* pv. *phaseolicola* (*Psph*) causes halo blight disease on beans. We discovered that the bicistronic genes in the *rhpPC* locus of *Psph* act coordinately to regulate the T3SS gene expression, bacterial fitness, and pathogenicity. *rhpP* encodes a metalloprotease that can degrade the key T3SS regulator protein HrpL and reduce bacterial fitness. *rhpC* encodes a chaperone that inhibits the RhpP activity and mediates translocation of RhpP to the periplasm. The level of *rhpP* RNA is high in KB but decreases in MM, but the *rhpC* RNA is low in KB but increases in MM. The elevated *rhpC/rhpP* transcript ratio in MM plus the inhibition of RhpC on RhpP activity in cytoplasm provide double insurance that warrants high induction of the T3SS genes in MM and bacterial fitness. The coordination between *rhpP* and *rhpC* reveals a new mechanism regulating bacterial pathogenicity, and may provide an important target for controlling bacterial pathogens.

## Introduction

Many Gram-negative bacterial pathogens rely on the T3SS for successful infection of their hosts [1]. The T3SS is encoded by a cluster of *hrp/hrc* genes that are essential for the induction of a hypersensitive response (HR) in resistant and nonhost plants and pathogenicity in susceptible plants [2]. The T3SS functions as a conduit to deliver an array of effector proteins into plant cells [3]. The effectors interfere with the host defense systems and contribute to bacterial pathogenicity [4]. Some effectors elicit HR and disease resistance in plants containing cognate disease resistance genes, and are therefore named avirulence proteins [5].

The expression of *hrp/hrc* genes and the effector genes (together called T3SS genes hereafter) is coordinately regulated by various environmental and host factors [6]. T3SS genes are expressed at a very low level when grown in nutrient-rich medium, but quickly induced to high levels after the bacteria are infiltrated into plants or cultured in nutrient-deficient medium that is believed to resemble the environment of the plant intercellular spaces where the bacteria proliferate during infection [7, 8].

As depicted in S1 Fig, the T3SS genes in *P*. *syringae* and *P*. *savastanoi* are activated by the extracytoplasmic function (ECF)-family alternate σ factor HrpL that recognizes a *hrp* box motif conserved in the promoters of many T3SS genes [9–11]. In turn, transcription of *hrpL* is controlled by a σ^54^-dependent promoter in an alternate σ factor RpoN-dependent manner [12]. Activation of *hrpL* also requires HrpR and HrpS, two homologous enhancer-binding proteins [13]. The *hrpR* and *hrpS* genes are in the same operon proceeded by the *hrpR* promoter [14]. HrpR and HrpS form a heterodimer that binds the *hrpL* promoter and induces *hrpL* transcription via interaction with the RpoN RNA polymerase holoenzyme [15,16].

The *hrpRS* operon is moderately expressed in KB and further induced in MM and in the plant [14–16]. A number of genes, including *hrpA*, *aefR*, and at least two two-component systems, *gacAS* and *rhpRS*, have been reported to regulate the transcription of the *hrpRS* operon [17–21]. *hrpA* encodes the T3SS pilus structural protein. Mutation of *hrpA* severely reduces the transcription of *hrpRS*, *hrpL* and T3SS genes [17]. AefR is a regulator of quorum sensing and epiphytic traits, and an *aefR* mutation reduces the *hrpR* promoter activity [18]. The GacAS system regulates multiple biological processes in various bacterial species, including motility, virulence, quorum sensing, and production of toxins, antibiotics, exopolysaccharides, and biofilms [19, 20]. A mutation in the response regulator gene *gacA* severely reduces the expression of *hrpRS* and the downstream cascade genes [20]. Thus far, the mechanisms by which HrpA, AefR, and GacAS regulate the *hrpRS* expression remain unknown. RhpRS was identified as a negative regulator of the T3SS genes [21]. The response regulator RhpR, upon phosphorylation, binds a putative inverted repeat motif in the *hrpR* promoter and represses *hrpRS* transcription [21, 22]. Dephosphorylation of RhpR reduces the binding affinity of RhpR to the *hrpR* promoter, and consequently the *hrpRS* promoter is de-repressed [21–23]. RhpS is a sensor kinase with dual activities. Under T3SS inducing conditions, RhpS presumably acts as a phosphatase that renders RhpR in the dephosphorylation state, which derepresses the *hrpRS* operon [21–23].

Another level of control of T3SS gene expression involves the stability of the HrpR protein, which is regulated by ATP-dependent protease Lon [24, 25]. In a *lon* mutant, HrpR is stabilized, which elevates the expression of downstream genes [24, 25].

The activity of HrpS is repressed by HrpV, a T3SS negative regulator that physically interacts with the HrpS protein [26]. HrpV-mediated repression can be suppressed by HrpG, a chaperone-like protein that interacts with HrpV [27]. The interaction of HrpG with HrpV liberates HrpS from the HrpV-repression and consequently up-regulates the T3SS cascade [27].

To investigate how *Pseudomonas* bacteria activate the T3SS genes in plants, a *P*. *savastanoi* pv. *phaseolicola* (*Psph*) mutant was isolated with diminished induction of *avrPto-luc* in *Arabidopsis*. We named the mutant gene *rhpC* (regulator of hrp, chaperone). *rhpC* is located immediately downstream of a putative metalloprotease gene that we called *rhpP* (regulator of hrp, protease). Here, we report the molecular and functional interactions between RhpP and RhpC in regulating the T3SS genes and bacterial fitness.

## Results

### Isolation of *rhpC*^−^ mutant in *Psph*

*avrPto* is a *Pseudomonas* T3SS effector gene carrying a typical *hrp*-box promoter [10]. In a previous study, we fused the *avrPto* promoter with the firefly luciferase gene *luc* in a broad host plasmid and constructed a reporter system for the T3SS gene expression in plants and MM [28]. To identify bacterial genes regulating the T3SS gene expression in plants, we constructed a transposon insertion mutant library in a *Psph* NPS3121 strain carrying the *avrPto-luc* reporter gene and screened for mutants with diminished *avrPto-luc* induction in *Arabidopsis att1* plants [18, 21]. *att1* was previously isolated by our group, and it supported several-fold higher induction of *avrPto* and *hrpL* promoters than did the wild type *Arabidopsis* [28]. *att1* plants provide a quick and sensitive reporter system for the assay of *Pseudomonas* T3SS gene regulation in plants [18, 21, 28]. The *Psph* NPS3121 strain is a non-host bacterium to *Arabidopsis* Col-0, and it does not multiply in the wild-type (WT) Col-0 and *att1* plants [28, 29]. Using the *avrPto-luc* reporter and *att1* plants, we previously isolated a number of T3SS regulatory genes in the *Psph* NPS3121 strain, including *aefR*, *rhpRS*, *hrpL*, *hrpR*, and *hrpS* [18, 21].

**Fig 1:**
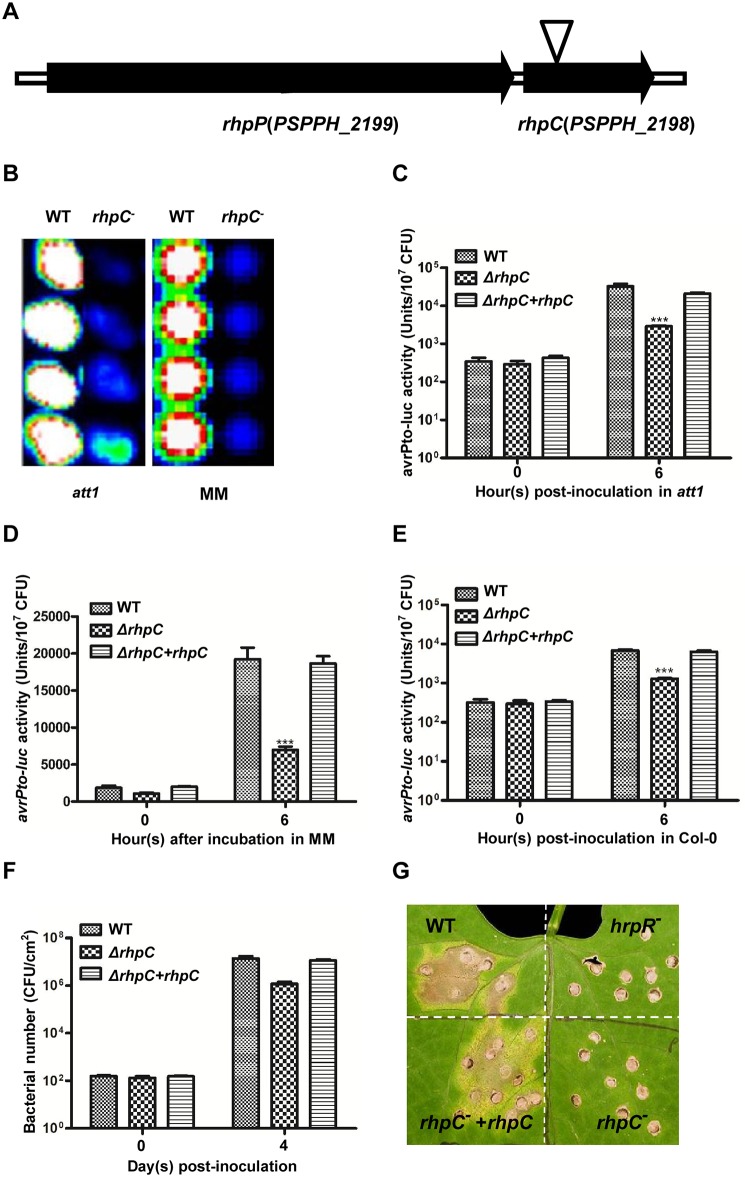
Impact of *rhpC* mutation on the T3SS gene induction and bacterial pathogenicity. (A) Gene organization and transposon insertion site in *rhpPC*. (B) Images captured by cooled charge-couple device showing the *avrPto-luc* activity 6 h after the WT *Psph* and the transposon-insertion mutant *rhpC*^-^ were infiltrated into the *Arabidopsis att1* plant (left) or cultured in MM (right). (C-E) Quantitative measurements of *avrPto-luc* activities in WT *Psph*, *ΔrhpC* mutant, and the complemented *ΔrhpC* mutant containing pML122::*rhpC-HA* after the bacteria were infiltrated into the *Arabidopsis att1* plant (C), cultured in MM (D), or infiltrated into Col-0 plants (E). The luciferase activity was measured at 0 and 6 h after induction, using a cooled charge-couple device, and was normalized by the bacterial number. Error bars indicate standard error. *** indicates the difference is statistically significant (p-value<0.001). The experiments were repeated at least five times with similar results. Bacterial growth (F) and disease symptoms (G) of the WT *Psph*, *ΔrhpC* mutant, and complemented *ΔrhpC* mutant in the host red kidney bean plants. Primary leaves of the bean plants were inoculated with 2x10^4^ CFU/ml of bacteria for bacterial growth assay and disease symptoms. Bacterial numbers were measured at 0 and 4 days after inoculation. Numbers represent the average of bacteria in four leaf disks. Error bars indicate standard error. Disease symptoms were photographed 7 days after inoculation. *hrpR*^-^ mutant was used as a reference to show the impact of *ΔrhpC* mutation on disease symptoms.

One *Psph* mutant we identified carried a transposon insertion in PSPPH2198 [30], which we later called *rhpC* because of its putative role as a molecular chaperone (Fig 1A). The mutant showed reduced induction of *avrPto-luc* not only in the *att1* plant but also in MM (Fig 1B). A deletion mutant of *rhpC* (*ΔrhpC*) was subsequently constructed, and like the transposon insertion mutant, *ΔrhpC* also showed a reduction of the *avrPto-luc* activities in *att1* and MM (Fig 1C and 1D). When inoculated into the wild-type *Arabidopsis* Col-0 plants, the *ΔrhpC* mutant also showed lower induction of *avrPto-luc* than did the wild-type strain (Fig 1E). The bacterial numbers of the wild-type *Psph* and the *ΔrhpC* mutant were similar and did not change clearly after inoculation into the Col-0 and *att1* plants (S2 Fig). Therefore, the reduced *avrPto-luc* activity in the *ΔrhpC* mutant reflected the poor induction of the reporter gene in the plants. When inoculated into host bean plants, the *ΔrhpC* mutant displayed reduced bacterial growth (Fig 1F) and disease symptoms (Fig 1G). The *avrPto-luc* induction in *Arabidopsis* plants and MM as well as the bacterial growth and disease symptoms in bean plants were complemented by a broad host plasmid with a constitutive promoter expressing the wild-type *rhpC* gene in the *ΔrhpC* mutant (Fig 1C–1G).

### Characteristics of the *rhpPC* locus

*rhpC* encodes a protein of 99 amino acid residues [30]. BlastP analysis identified homologous proteins of unknown function in a wide range of bacterial species (S3 Fig). A cytoplasmic localization of the RhpC protein was predicted by both CELLO [31, 32] and PredictProtein (https://predictprotein.org/). This small protein has an acidic isoelectric point (pI = 4.73) and predominantly *β*-sheet and α-helical secondary structures (S4 Fig).

*rhpC* is 20 bp downstream of PSPPH2199 (Fig 1A). PSPPH2199 encodes a protein of 356 amino acid residues [30]. BlastP analysis indicated that PSPPH2199 belongs to the metalloprotease of the M4 family [33]. We therefore named PSPPH2199 as RhpP.

RhpP homologues exist not only in *Pseudomonas* (S5 Fig) but also in a wide range of other bacteria (S6 Fig). Proteins of this family have three Zn^2+^-binding amino acid residues, including two histidine residues in the HEXXH motif and a unique glutamate residue toward the C-terminal from the motif [33]. Protein alignment indicated that these residues (His_176_, His_180_ and Glu_200_) were all conserved in RhpP (S7 and S8 Figs). Additional residues at the RhpP active site were 146, 147, 173, 177, 191, 240, 241, and 279 (S7 and S8 Figs). Interestingly, all bacterial strains, except one (*Desulfomonile tiedjei* DSM 6799), that carry the RhpC homologue also carry the RhpP homologue (S5, S6 and S8 Figs), and the two genes are arranged in the same manner as in the *Psph* strain, suggesting co-evolution of the *rhpPC* locus. However, many bacterial species, including the plant bacterial pathogens *Xanthomonas* and *Pectobacterium*, carry only the RhpP homologue without RhpC (S5 and S6 Figs).

### Differential expression of the *rhpPC* locus

**Fig 2:**
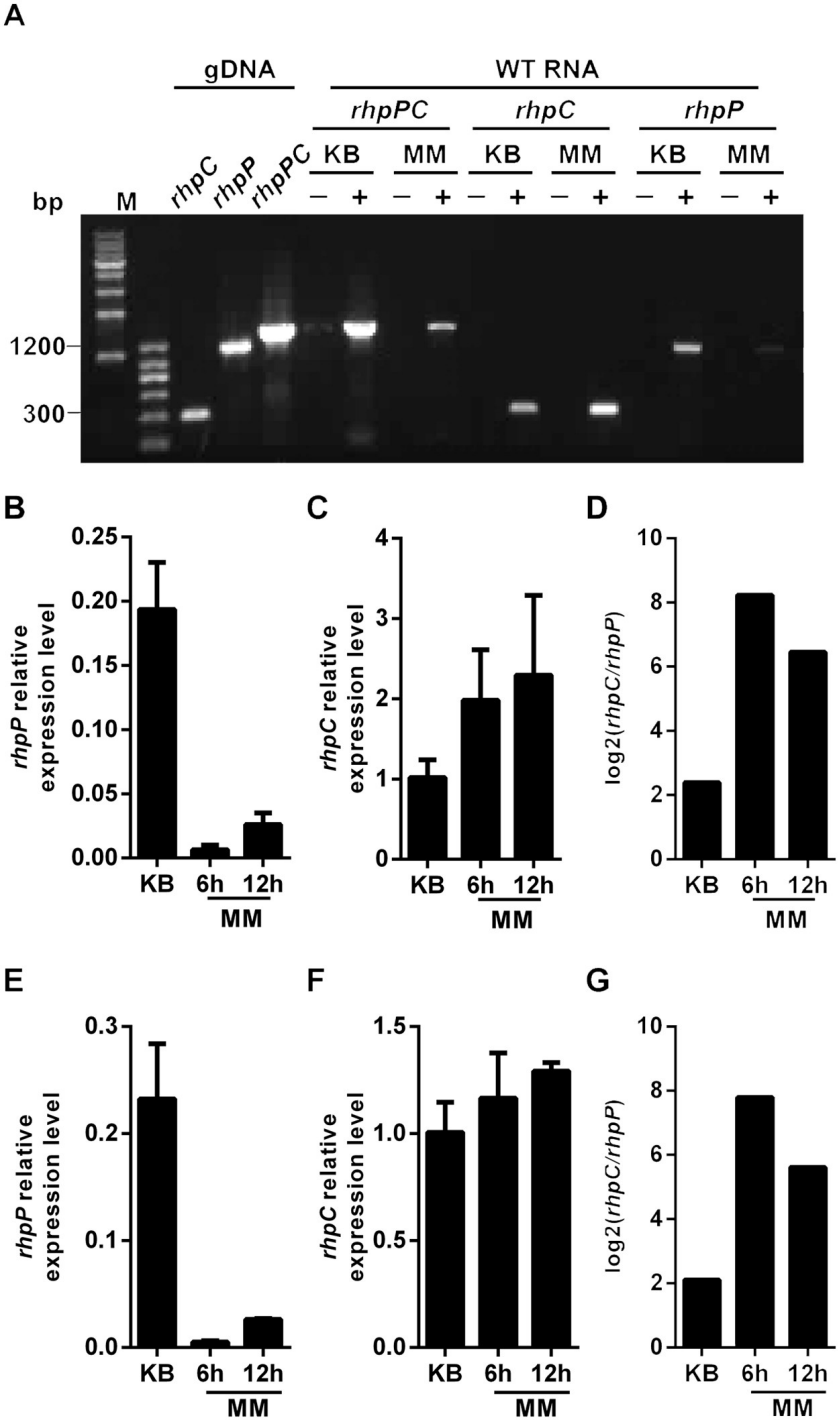
*rhpP and rhpC* are polycistronic and differentially expressed in KB and MM. (A) Total RNAs (2 μg) extracted from *Psph* bacterial cells grown in KB and MM were reverse-transcribed with the primer corresponding to the 3’ end of *rhpC*. 10% of each reaction was used as template for PCR. Primers for PCRs were shown in S9 Fig. Bacterial genomic DNA (gDNA) was used as control to show the size of each PCR product. “+” and “-” indicated PCR templates from reactions with and without reverse transcriptase, respectively. 35 cycles were used for RT-PCR of *rhpPC* and *rhpP*, and 25 cycles were used for RT-PCR of *rhpC*. M indicates DNA molecular markers. (B-G) Real time PCR analyses of *rhpP* and *rhpC* RNA expression in *Psph* bacterium grown in KB and MM. Total RNAs were reverse-transcribed using random primers. The reactions were first treated with RNase and then used as template for real-time PCR analyses. The relative expression levels of *rhpP* and *rhpC* were normalized to 16S *rRNA* (B-C) and *rpoD* (E-F) transcripts, respectively. (D) and (G) show the ratio of *rhpC*/*rhpP* RNAs in each sample.

The close proximity of *rhpC* and *rhpP* genes in the bacterial genome led us to test whether the two genes are co-transcribed into a polycistronic RNA. Total RNA was extracted from the NPS3121 strains cultured in KB and MM and reverse-transcribed with a primer derived from the 3' end of *rhpC* (S9 Fig). RT-PCR analysis using one primer derived from the 5' end of *rhpP* and another primer derived from the 3' end of *rhpC* (S9 Fig) produced a DNA fragment of the expected size (Fig 2A), indicating that the two genes are transcribed into a polycistronic RNA. The same RNA samples were also reverse-transcribed using primers specific for *rhpC* and *rhpP*, respectively. RT-PCR with *rhpC-* and *rhpP*-specific primers also detected the respective transcripts (Fig 2A).

The different intensities of the RT-PCR products from the KB and MM cultures suggested possible differential regulation of *rhpC* and *rhpP* in response to different nutrient conditions (Fig 2A). To test this possibility, real-time PCR was performed to analyze the levels of *rhpP* and *rhpC* transcripts after reverse-transcription of the RNA samples with random primers, using 16S *rRNA* and *rpoD* as internal references. The *rhpP* RNA was more abundant in KB than in MM (Fig 2B and 2E), and *rhpC* showed the opposite expression patterns compared to *rhpP* (Fig 2C and 2F). The ratio of the *rhpC*/*rhpP* transcripts in KB was much lower than the ratio in MM (Fig 2D and 2G), suggesting that *rhpP* and *rhpC* were expressed not only as polycistronic RNA, but also in separate forms. The transcriptions of *rhpP* and *rhpC* were likely controlled by different promoters that were differentially regulated by nutrient conditions.

### Deletion of *rhpP* diminished the negative effect of *rhpC* mutation on the T3SS gene induction and bacterial virulence

**Fig 3:**
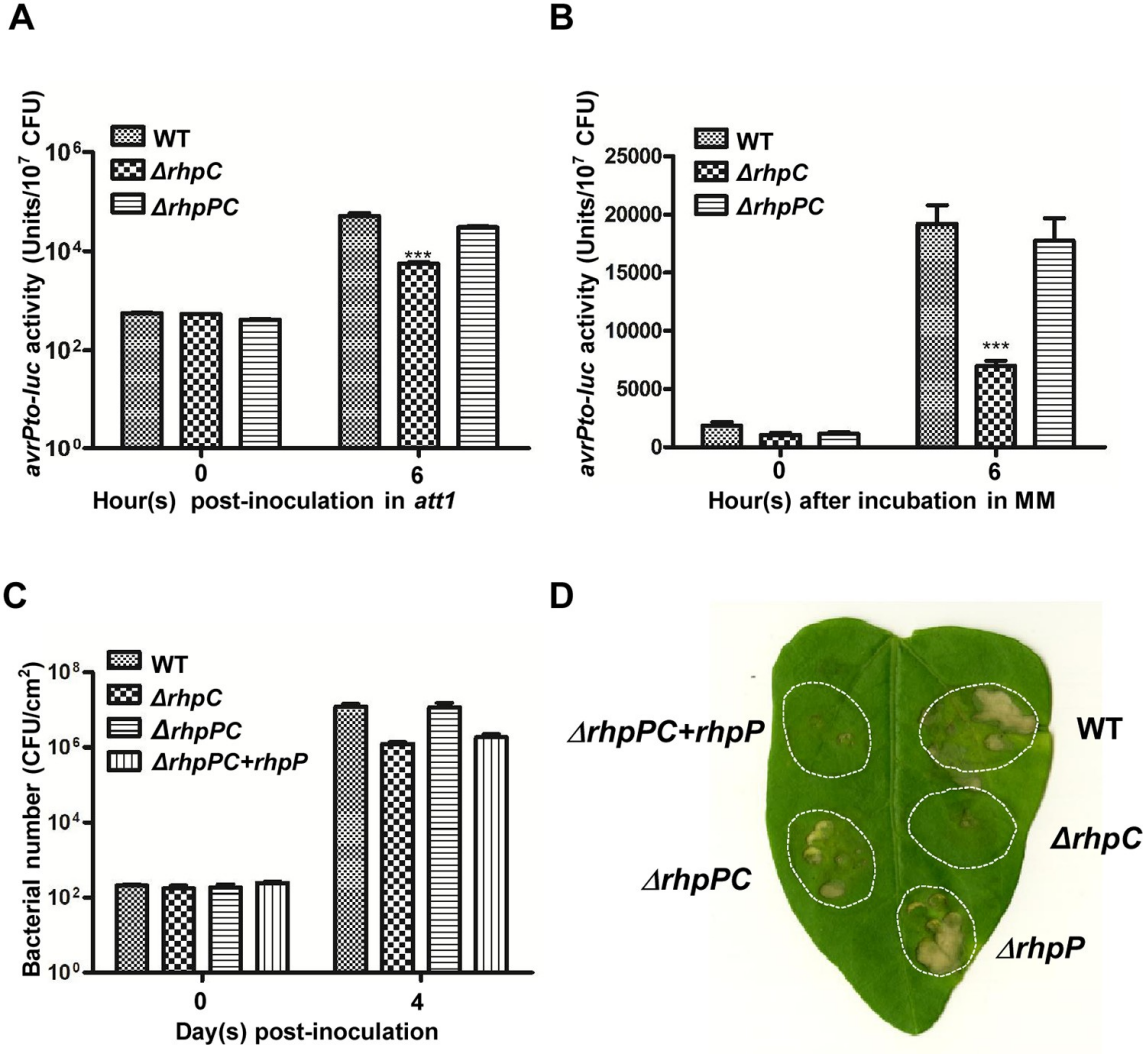
RhpP inhibited the T3SS gene induction and bacterial pathogenicity in the absence of RhpC. (A) and (B) WT *Psph*, *ΔrhpC* mutant, and *ΔrhpPC* mutant were infiltrated into the *Arabidopsis att1* plant (A) or cultured in MM (B). The luciferase activities were measured at 0 and 6 h after induction and normalized by the bacterial number. Error bars indicate standard error. *** indicates the difference is statistically significant (p-value<0.001). (C) WT *Psph*, *ΔrhpC* mutant, *ΔrhpPC* mutant, and *ΔrhpPC* mutant carrying the pHM1::*rhpP* plasmid (all at 2x10^4^ CFU/ml) were infiltrated into the primary leaves of bean plants. Bacterial numbers were measured at 0 and 4 days after inoculation. Numbers represent the average of bacteria in four leaf disks. Error bars indicate standard error. (D) Disease symptoms caused by the WT *Psph*, *ΔrhpC*, *ΔrhpP*, *ΔrhpPC*, and *ΔrhpPC* mutant carrying the pHM1::*rhpP* plasmid. The bacteria at 10^5^ CFU/ml were infiltrated into the primary leaves of bean plants. Disease symptoms were photographed 7 days after inoculation.

Polycistronic genes are often functionally related. To test whether *rhpP* and *rhpC* are related in function, deletion mutants of the whole *rhpPC* locus and *rhpP* were created by homologous recombination. The resulting mutants were tested for *avrPto-luc* induction and bacterial virulence. Compared to the *ΔrhpC* mutant, the *ΔrhpPC* strain displayed better induction of *avrPto-luc* in both the *att1* plants (Fig 3A) and MM (Fig 3B). In the host bean plants, the *ΔrhpPC* mutant displayed better bacterial growth and stronger disease symptoms than did the *ΔrhpC* mutant (Fig 3C and 3D). However, bacterial growth and disease symptoms in the host plants were repressed when a plasmid-borne *rhpP* under a constitutive promoter was expressed in the *ΔrhpPC* mutant (Fig 3C and 3D). Deletion of *rhpP* alone did not significantly affect the bacterial virulence, as shown by the similar disease symptoms induced by the wild-type strain and the *ΔrhpP* mutant in the host bean plants (Fig 3D). These results suggested that RhpP, when present alone in *Psph*, compromised the induction of the T3SS genes and bacterial virulence, and the presence of RhpC repressed the negative effect of RhpP.

### RhpC is a cytoplasmic protein required for translocation of RhpP to periplasm

**Fig 4:**
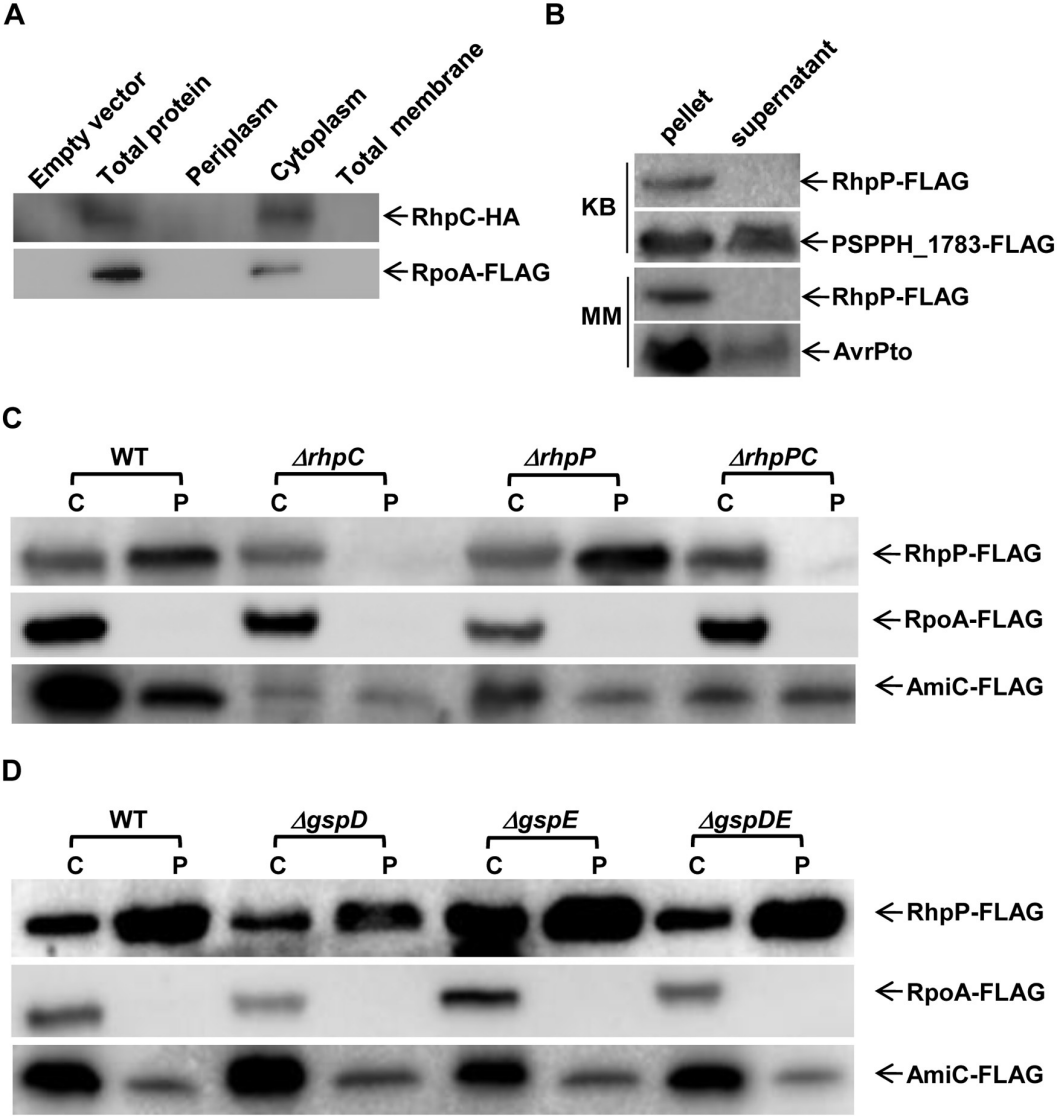
Localization assay of RhpC and RhpP proteins. (A) Localization of RhpC. Bacterial cells expressing RhpC-HA was fractionated into periplasm, cytoplasm, and membrane fractions. The presence of RhpC protein was determined by Western blot analysis using anti-HA antibody. RopA was used as cell fractionation control. (B) Secretion assay of RhpP in the wild type *Psph* strain. The wild type *Psph* strain carrying pHM1::*rhpP-FLAG* was cultured in 500 mL liquid KB or MM. The culture media were separated from the bacterial cells, and then precipitated with TCA. Protein in the TCA-precipitation and bacterial cells were assayed by immuno-blotting using anti-FLAG antibody. PSPPH_1783 and AvrPto were used as controls for protein out-secretion in KB and MM, respectively. (C) RhpC is required for RhpP translocation to the periplasm. Wild type *Psph*, *ΔrhpC*, *ΔrhpP*, and *ΔrhpPC* mutant strains carrying pHM1::*rhpP-FLAG* were grown in KB. Periplasmic and cytoplasmic proteins were separated and assayed using Western blot with anti-FLAG antibody. Cytoplasmic protein RpoA and periplasmic protein AmiC were used as cell fractionation control. C, cytoplasmic protein; P, periplasmic protein. (D) Mutations of *gspD* and *gspE* genes did not block the translocation of RhpP to periplasm. Cytoplasmic RpoA and periplasmic AmiC through the Tat pathway were used as cell fractionation control. C, cytoplasmic protein; P, periplasmic protein.

The sequence of *rhpC* predicts a cytoplasmic protein. To test this, we tagged RhpC at the C-terminus with HA and expressed the gene in the *ΔrhpC* mutant strain using a broad host plasmid. Bacterial protein was separated into periplasmic, membrane, and cytoplasmic fractions. Western blot analysis indicated that RhpC, like the cytoplasmic control protein RopA [34], was exclusively localized in the cytoplasm (Fig 4A).

Metalloproteases of the M4 family in bacterial pathogens are largely secreted to the extracellular milieu by their host bacteria [35]. We therefore examined whether RhpP was also a secreted protein. RhpP was FLAG-tagged at the C-terminus in the broad host vector pHM1 under a constitutive promoter and expressed in the wide-type *Psph* strain. The resulting bacterium was cultured in KB and MM. The bacterial cells and culture media were then separated by centrifugation, the protein in the culturing media was precipitated by TCA, and the presence of RhpP-FLAG was examined using the anti-FLAG antibody. PSPPH_1783, which is homologous to the *P*. *aeruginosa* out-secreted protein PlcN/PlcH through the type II secretion pathway, was used as the secretion control in KB [36]. The T3SS effector AvrPto was used as a secretion control in MM [37]. Unlike the control proteins that were detected in the culture media, no RhpP-FLAG protein was detected in KB or MM, even when 500 mL of culture medium was used for protein precipitation (Fig 4B), indicating that RhpP was not secreted to the outside of the bacterial cells at the tested conditions.

Sequence analysis of RhpP did not predict a membrane localization signal. The other possible compartment for RhpP localization would be the cytoplasm or periplasm. We therefore separated the bacterial cells into periplasmic and cytoplasmic fractions using osmotic shock. AmiC, a protein that is dispersed throughout the new-born cells and is secreted to the periplasm in divisional cells [38], was used to indicate the protein fractionation in periplasm. The cytoplasmic protein RpoA [34] was used as control of protein fractionation in cytoplasm. Western blot analysis indicated that the RhpP protein was present in both the cytoplasm and the periplasm in the WT strain (Fig 4C).

As RhpC inhibited the function of RhpP, we asked whether RhpC affects the localization of RhpP. We therefore introduced the *pHM1*::*rhpP-FLAG* plasmid into *ΔrhpP*, *ΔrhpC*, and *ΔrhpPC* mutants and examined the RhpP localization. RhpP was present in both the periplasm and the cytoplasm of the *ΔrhpP* mutant, but was detected only in the cytoplasm of the *ΔrhpC* and *ΔrhpPC* mutants (Fig 4C), suggesting that RhpC is required for the translocation of RhpP to the periplasm.

We further tested whether RhpP translocation to the periplasm was through the general secretion pathway. As shown in Fig 4D, mutations of *gspD* and *gspE* genes of the general secretion pathway did not affect the RhpP localization in the *Psph* strain.

### RhpC physically interacts with RhpP and stabilizes RhpP in *Psph*

**Fig 5:**
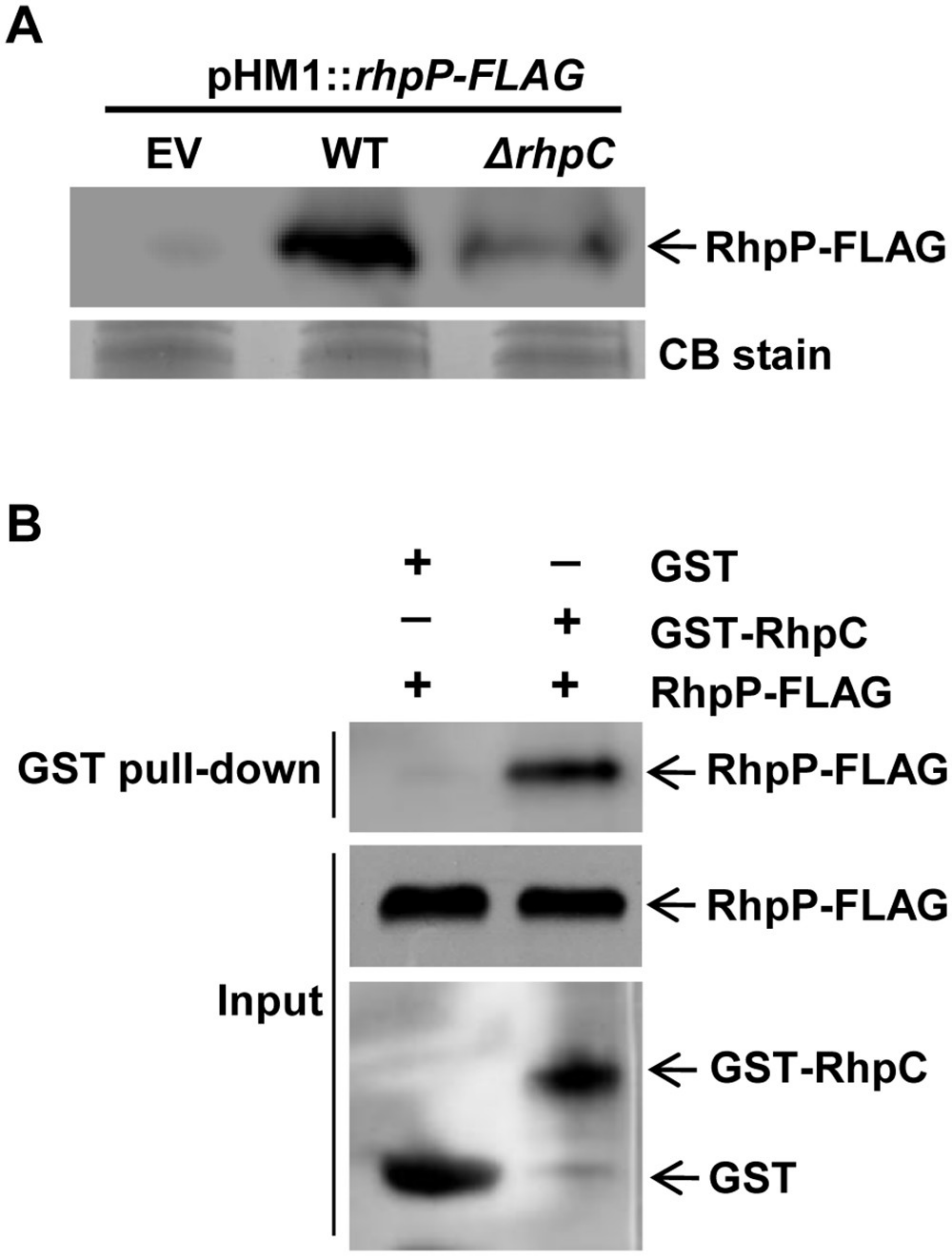
RhpC interacted with and stabilized RhpP protein. (A) Wild type *Psph* and *ΔrhpC* mutant constitutively expressing RhpP-FLAG were cultured in KB. The total protein in bacterial cells was examined using Western blot with anti-FLAG antibody. *Psph* strain carrying the empty pHM1 vector (EV) was used as control. (B) Purified GST or GST-RhpC coupled with the Glutathione Sepharose beads was incubated with the purified RhpP-FLAG protein and then precipitated by centrifugation. Protein pulled down by the beads was assayed using Western blot with anti-FLAG antibody. CB stain, Coomassie Blue stain.

The *rhpP-FLAG* gene expressed by the pHM1 plasmid under a constitutive promoter produced stronger protein signal in the WT *Psph* strain than in the *ΔrhpC* mutant (Fig 5A), suggesting that RhpC stabilized RhpP in the bacterium. This result and the functional interactions between RhpC and RhpP in regulating the T3SS genes and bacterial virulence raised the question whether RhpC physically interacts with RhpP. We tested this using the protein pull-down assay. RhpP-FLAG was expressed in the WT *Psph* strain using a plasmid and purified using anti-FLAG affinity gel. The *E*. *coli* BL21 strain expressing RhpC-GST or GST was sonicated, and the protein was purified with glutathione sepharose. RhpC-GST or GST on glutathione sepharose was further incubated with the RhpP-FLAG protein eluted from the anti-FLAG affinity gel. Western blot analysis showed that RhpP-FLAG was specifically pulled down by RhpC-GST (Fig 5B), indicating the physical interaction between RhpP and RhpC.

### The presence of RhpP alone reduces the accumulation of HrpL but not HrpR and HrpS proteins in *Psph*

**Fig 6:**
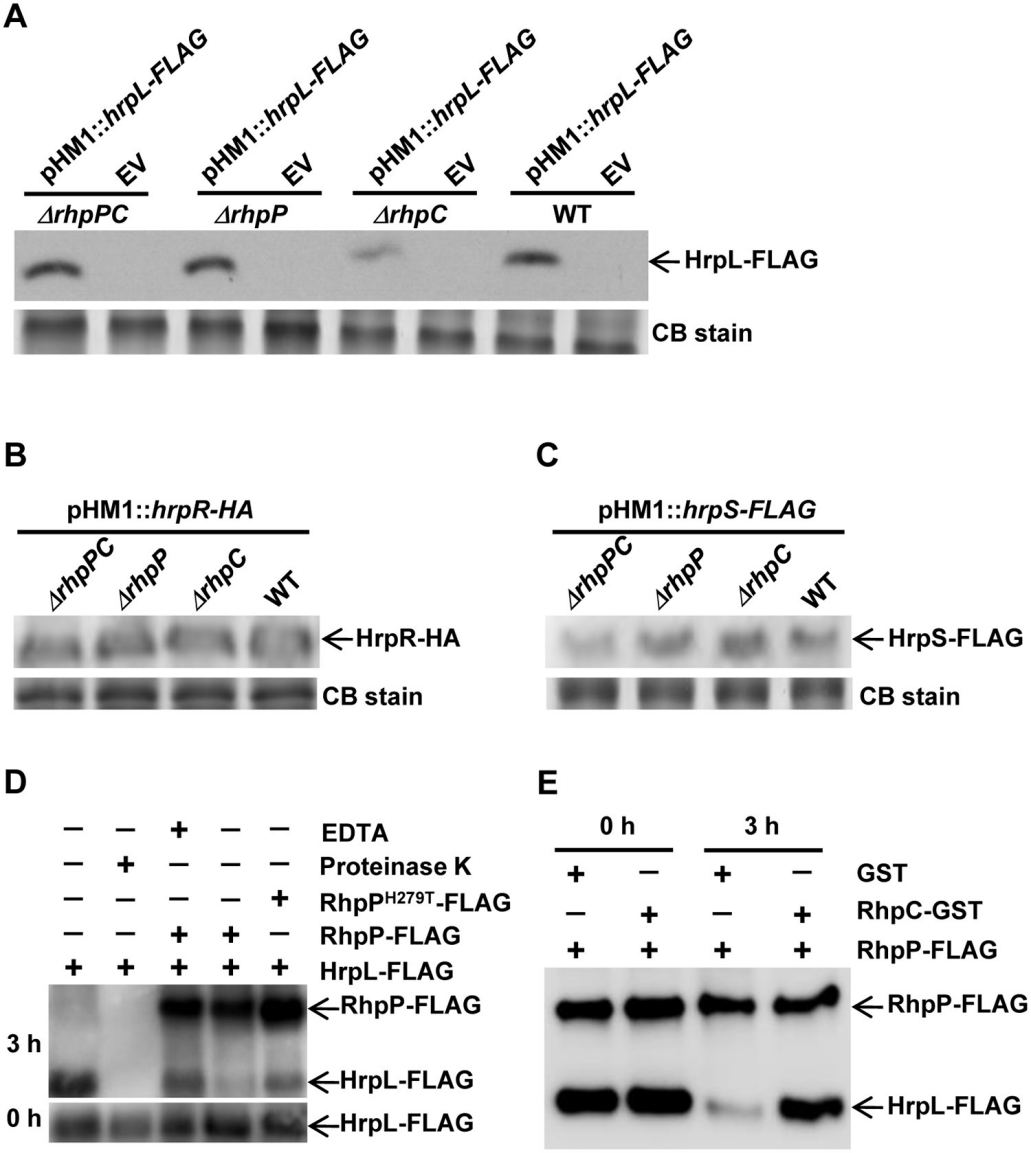
RhpP alone in *Psph* decreased the protein level of HrpL but not HrpR and HrpS. (A-C) HrpL-FLAG, HrpR-HA, and HrpS-FLAG were expressed in *Psph*, *ΔrhpC*, *ΔrhpP*, and *ΔrhpPC* strains using the broad host pHM1 plasmid under a constitutive promoter. The HrpL-FLAG (A), HrpR-HA (B), and HrpS-FLAG (C) protein levels were examined using Western blot with anti-FLAG and anti-HA antibodies. Empty pHM1 plasmid (EV) was also introduced into the bacterial strains as control. (D) *In vitro* digestion of HrpL protein by RhpP. RhpP-FLAG and RhpP^H279T^-FLAG proteins produced by the pHM1 plasmid were purified from the *Psph* strain. HrpL-FLAG protein produced by the pHM1 plasmid was purified from *E*. *coli* BL21 strain. The purified HrpL-FLAG was incubated with RhpP-FLAG, RhpP-FLAG plus EDTA, and RhpP^H279T^-FLAG in PBS buffer at 28°C for 3 h, and then examined by Western blot analysis. HrpL-FLAG incubated with protease K or PBS buffer alone was used as control. 0 h shows the initial level of HrpL protein in each reaction. (E) Inhibition of the RhpP-mediated degradation of HrpL by RhpC. The purified HrpL-FLAG was incubated with RhpP-FLAG plus purified RhpC-GST or GST for 0 and 3 h, and then examined by Western blot analysis with anti-FLAG antibody. CB stain, Coomassie Blue stain.

Because RhpP is a putative metalloprotease, we hypothesized that, in the absence of RhpC, RhpP represses the T3SS gene expression by destabilizing the TTSS regulatory proteins. To test this, we compared the levels of three major T3SS regulatory proteins, HrpL, HrpR, and HrpS, in the WT, *ΔrhpC*, *ΔrhpP*, and *ΔrhpPC* strains. All these proteins were expressed under a constitutive promoter in the pHM1 plasmid. Western blot analysis revealed a lower level of HrpL protein in the *ΔrhpC* mutant than in other strains (Fig 6A), which was consistent with the lower T3SS gene expression in this strain. The levels of HrpR protein were similar in all the strains (Fig 6B), and so were the levels of HrpS protein (Fig 6C).

The reduced level of HrpL protein in the *ΔrhpC* mutant might have resulted from degradation of HrpL by RhpP. To test this possibility, we purified HrpL-FLAG and RhpP-FLAG using anti-FLAG affinity gel. The purified proteins were incubated together in PBS buffer, and the levels of HrpL-FLAG protein were examined after 3 h of incubation. In the presence of PBS buffer only, HrpL-FLAG was stable (Fig 6D). However, the level of HrpL-FLAG was significantly reduced after incubation with RhpP-FLAG (Fig 6D). The reduction of HrpL-FLAG was much suppressed when EDTA, a metalloprotease inhibitor, was added with RhpP-FLAG, or when HrpL-FLAG was incubated with RhpP^H279T^, a RhpP mutant of the protease active site (Fig 6D). These results indicated that RhpP was an active metalloprotease for HrpL. To test whether RhpC had any effect on the RhpP protease activity, RhpC was added to the reaction. Co-incubation of RhpC with RhpP stabilized the HrpL-FLAG protein (Fig 6E), indicating that RhpC blocked the RhpP protease activity.

### The detrimental effect of RhpP to bacterial fitness requires active enzymatic sites and is suppressed by RhpC

**Fig 7:**
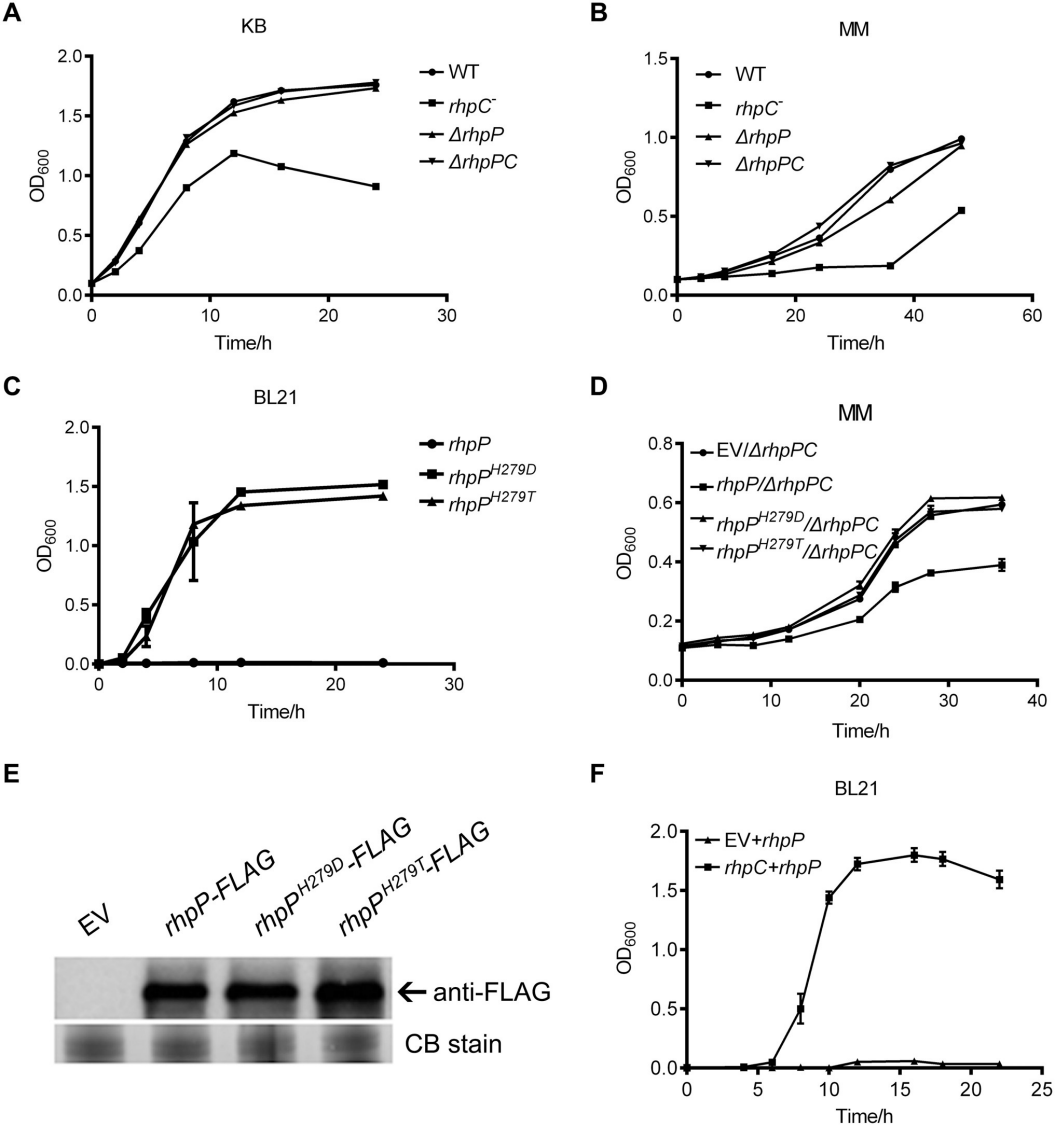
The detrimental effect of RhpP on bacterial growth was abolished by mutations of protease active sites and inhibited by RhpC. (A) and (B) Growth curves of the wild type *Psph*, *ΔrhpC*, *ΔrhpP*, and *ΔrhpPC* strains in KB (A) and MM (B). (C) Growth curves of *E*. *coli* BL21 strains expressing the wild type RhpP-MBP and the mutant RhpP^H279D^-MBP and RhpP^H279T^-MBP proteins. (D) Growth curves of *ΔrhpPC* mutant strains carrying the empty pHM1 vector or pHM1 expressing RhpP-FLAG, RhpP^H279D^-FLAG, and RhpP^H279T^-FLAG proteins in MM. (E) RhpP-FLAG, RhpP^H279D^-FLAG, and RhpP^H279T^-FLAG proteins expressed by the pHM1 plasmid in *ΔrhpPC* mutant. Equal amounts of bacterial cells were boiled in SDS sample buffer and analyzed using Western blot with anti-FLAG antibody. (F) Growth of the BL21 strain carrying pMAL::*rhpP-MBP* and pHM1 empty vector, or carrying pMAL::*rhpP-MBP* and pHM1::*rhpC* in LB. EV, empty vector; CB stain, Coomassie Blue stain.

We consistently observed smaller colonies of the *ΔrhpC* mutant than the WT, *ΔrhpP*, and *ΔrhpPC* strains on the KB plate. In liquid KB or MM culture, the *ΔrhpC* mutant also displayed slower growth, compared to the other three strains (Fig 7A and 7B). When RhpP was tagged with MBP in the pMAL-p_2_X plasmid and transformed into the *E*. *coli* BL21 strain, the resulting colonies were very small on the LB plate. Liquid LB medium inoculated with these small colonies stayed clear, even after 24 h of culture (Fig 7C). These results indicated that RhpP, when presented alone, was detrimental not only to the native bacterium but also to other bacterial species.

To determine whether the detrimental effect of RhpP was related to the protease activity, amino acid residue His_279_ at the putative active site, was mutagenized to Asp and Thr. *E*. *coli* BL21 strains expressing the mutant recombinant proteins (RhpP^H279D^-MBP and RhpP^H279T^-MBP) showed normal growth in liquid LB culture (Fig 7C). The RhpP^H279D^ and RhpP^H279T^ proteins were also compared to RhpP when expressed in the *ΔrhpPC* strain. Unlike RhpP, neither RhpP^H279D^ nor RhpP^H279T^ affected the bacterial growth (Fig 7D; S10 Fig). Western blot analysis indicated that the RhpP^H279D^ and RhpP^H279T^ proteins were as stable as RhpP in the *ΔrhpPC* strain (Fig 7E). These results suggested that the negative effect of RhpP on bacterial growth was mediated by the protease activity.

As RhpC inhibited the RhpP protease activity, and the *Psph* strain harboring the *rhpPC* locus was normal, we speculated that the presence of RhpC could repress the detrimental effect of RhpP on bacterial growth. This possibility was further tested by the co-expression of RhpC and RhpP in the *E*. *coli* BL21 strain. pMAL-p_2_X plasmid expressing RhpP-MBP, when introduced alone into BL21, severely inhibited the bacterial growth. However, when this plasmid was introduced into the BL21 strain expressing RhpC, the resulted bacterium grew normally both on the LB plate and in the LB liquid medium (Fig 7F). These results suggested that RhpC suppressed the detrimental effect of RhpP on bacterial fitness, probably by inhibiting the protease activity of RhpP in the cytoplasm.

### RhpP mutants of the protease active sites retain the RhpC interaction and are capable of periplasmic translocation

**Fig 8:**
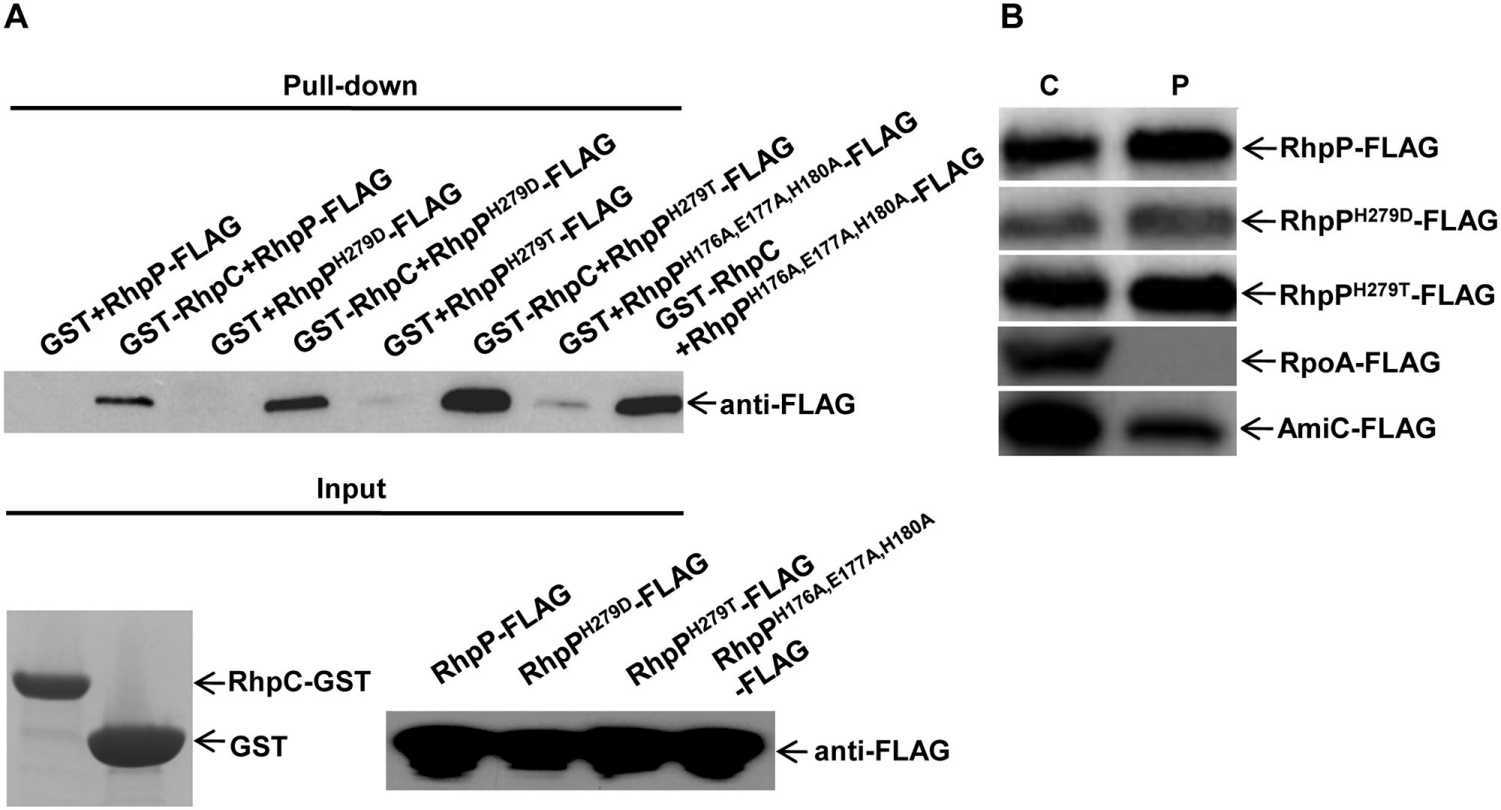
Mutants of RhpP active sites retained interaction with RhpC and translocation to periplasm. (A) Interaction of RhpC with RhpP mutants. RhpP and RhpP mutants of the active sites (RhpP^H279D^, RhpP^H279T^, and RhpP^H176A,E177A,H180A^) were tagged with FLAG and expressed in the wild type *Psph* strain using pHM1 plasmid. The purified proteins were incubated with the purified GST or GST-RhpC on the glutathione sepharose beads and then precipitated by centrifugation. Protein pulled down by the beads were assayed using Western blot with anti-FLAG antibody. (B) Translocation of RhpP mutants. RhpP and RhpP mutants of the active sites (RhpP^H279D^, RhpP^H279T^) were tagged with FLAG and expressed in the wild type *Psph* strain using the pHM1 plasmid. Proteins in the periplasm and cytoplasm were examined using Western blot with anti-FLAG antibody. Cytoplasmic protein RpoA and periplasmic protein AmiC were used as protein fractionation control. C, cytoplasmic protein; P, periplasmic protein.

Given that RhpC interacts with RhpP and inhibits its protease activity, we wondered whether mutation of the active sites of RhpP interferes with the RhpC interaction. This was tested by protein pull-down assay between GST-RhpC and RhpP mutant proteins. In addition to RhpP^H279D^ and RhpP^H279T^, we created another mutant with the three conserved zinc-binding residues all changed to Ala. As shown in Fig 8A, all the mutant proteins were pulled down specifically by GST-RhpC, indicating that the mutations of the active sites did not interrupt the interaction with RhpC. We also tested whether the mutations affected the RhpP protein translocation to the periplasm. As shown in Fig 8B, the mutant proteins, like the wild type RhpP, were also translocated to the periplasm.

## Discussion

In the search for genes regulating the T3SS genes in *Psph*, we identified the mutant of the *rhpC* gene with diminished T3SS gene induction and reduced pathogenicity in the host plant. *rhpC* is downstream of *rhpP* which encodes a putative metalloprotease. RhpC is a cytoplasmic protein that interacts with RhpP and facilitates the translocation of RhpP to the periplasm. The expression of RhpP alone in *Psph* and *E*. *coli* inhibited bacterial growth, and the inhibition was abolished by mutation of the conserved protease activity sites, suggesting that RhpP alone is an active protease in the bacterial cytoplasm. Purified recombinant RhpP protein can degrade HrpL *in vitro*, further showing RhpP as an active protease. The *ΔrhpC* mutant exhibited a reduced level of HrpL protein, the direct regulator of the T3SS genes. The reduction in HrpL was consistent with the reduced T3SS gene induction in the *ΔrhpC* mutant. Interestingly, the *ΔrhpC* mutant did not show a reduction in the HrpS and HrpR protein levels, suggesting that RhpP has substrate specificity. Nonetheless, HrpL is unlikely to be the only substrate for RhpP in the *Psph* cytoplasm, because *hrpL*^-^ mutant did not show any growth defect in KB and MM [13, 39]. In addition, RhpP probably degraded the *E*. *coli* proteins as well because expression of the RhpP-MBP recombinant protein in *E*. *coli* caused a severe growth defect, and the detrimental effect in *E*. *coli* required the protease active sites of RhpP. The reduced growth of the *ΔrhpC* mutant in the host plants was likely the result of reduced T3SS gene expression plus the reduced fitness of the bacterial cells.

Protein localization analysis revealed that, in the WT *Psph* strain, an RhpP protein of the same size was present in the cytoplasm and the periplasm but not in the culture medium. This is different from the known RhpP homologues such as thermolysin from *Bacillus thermoproteolyticus* [40], elastase from *Pseudomonas aeruginosa* [41], Prt1 in *Pectobacterium cratovorum* [42], and many other M4 metalloproteases from other bacteria, which are all secreted proteins to extracellular milieu [35, 43–45]. These extracellular proteases are encoded as inactive preproproteases with an N-terminal signal peptide that is followed by a propeptide and a catalytic domain. The signal peptides mediate the translocation of these preproproteases through the Sec pathway to periplasm, where the signal peptides are removed by the periplasmic-localized signal peptidase [35, 43]. Immediately following the signal peptides are propeptides which are also called intramolecular chaperones [35, 43]. Propeptides assist in the folding of the catalytic domains into active conformation [44, 45]. Upon the completion of protein folding, propeptides are cleaved either by autoprocessing or with the help of other proteases [44]. The cleaved propeptide stays in contact with the catalytic domain, which stabilizes the substrate protein, prevents premature activation of the protease, and facilitates translocation of the protease through the outer membrane [46, 47]. Alignment of RhpP with the out-secreted M4 metalloproteases showed a conserved C-terminal catalytic domain preceded by an N-terminal peptide of 67 amino acid residues. SignalP analysis did not find a signal peptide for the Sec pathway in the N-terminal sequence (S11 Fig). The propeptide sequences presented in the known M4 metalloproteases were not found in RhpP (S7 Fig). Consistently, mutations of the general secretion pathway genes *gspD* and *gspE* did not block the translocation of RhpP to the periplasm.

Characterization of *rhpC* led us to propose that RhpC is a specific chaperone for RhpP. First, in almost all sequenced bacterial genomes carrying an RhpC homologue, the *rhpC* homologous gene is always coupled by the *rhpP* homologous gene, suggesting co-evolution of the two genes. Second, RhpC is a small cytoplasmic protein (molecular mass = 11 kDa) with a relatively low isoelectric point and a C-terminal helical structure. These characteristics are also shared by many other chaperones [48, 49]. In addition, RhpC is required for RhpP protein stability and translocation from the cytoplasm to the periplasm. The presence of RhpC inhibited the RhpP protease activity and the detrimental effect of RhpP on bacterial fitness, suggesting that RhpC is required for the proper function of RhpP. The interaction of RhpC with RhpP in the protein pull-down assay provided further evidence that RhpC is the chaperone of RhpP. Based on these characteristics, RhpC appears to be a functional mimic of prepropeptides of the out-secreted metalloproteases. Although RhpC can inhibit the protease activity of RhpP, mutations of the protease active sites (His_176_, Glu_177_, His_180_, and His_279_) in RhpP did not abolish the interaction with RhpC, suggesting that these active sites are probably not at the RhpC-interacting surface. These RhpP mutant proteins were also translocated to the periplasm in the presence of RhpC. It is possible that the RhpC interaction changes RhpP to an inactive conformation that is compatible with the translocation through the bacterial inner membrane.

Gram-negative bacteria have five classes of protein secretion systems spanning both the inner membrane and the outer membrane that deliver cytoplasmic proteins to the outside of the bacterial cell, including type I secretion system (T1SS), T2SS, T3SS, T4SS, and T6SS [50]. However, only two classes of protein transporters have been reported that span the inner membrane and deliver proteins to the periplasm, including the Sec apparatus and the Tat system (twin-arginine translocation system) [50, 51]. The involvement of the Sec pathway in RhpP translocation was ruled out because deletion of *gspE* (*secE*) did not block the RhpP translocation to the periplasm. Proteins targeting to the Tat machinery usually have signal peptides that contain a conserved twin-arginine motif [51]. TatP analysis predicted a low Tat-targeting signal and a peptide cleavage site between A_49_ and R_50_ in the N-terminus of RhpP (S12 Fig). However, the same molecular weight of RhpP detected by Western blot in cytoplasm and periplasm did not support the cleavage of the signal peptide. Because RhpC interacts with RhpP and is required for RhpP translocation, we wondered if RhpC has a signal peptide for the Tat system and carries RhpP to the Tat system in a "piggy-back" manner. However, we did not find the twin-arginine signal peptide in RhpC. Instead, we found significant similarity between RhpC and TatB of the Tat system (S13 Fig). The functional significance of this sequence similarity is unknown. Whether RhpP passes through the cytoplasmic membrane through the Tat system remains to be investigated.

Real-time PCR analysis indicated that the *rhpPC* locus is subjected to complex regulation in response to nutrient conditions. This locus is transcribed not only into *rhpPC* polycistronic RNA but also into discrete *rhpC* RNA. The *rhpP* (or *rhpPC*) RNA is more abundant in KB than in MM, opposite to *rhpC* that is expressed at a higher level in MM than in KB. The ratio of *rhpC*/*rhpP* transcripts is also much higher in MM than in KB. Given the facts that RhpP in cytoplasm can degrade the HrpL protein and that the RhpP protease activity can be repressed by RhpC, we propose that the reduced *rhpP* transcripts and induced *rhpC* transcripts in MM serve as double protection that restricts the detrimental RhpP activity to a minimal level in the cytoplasm, which in turn secures the high induction of the T3SS genes. It is worthwhile to mention that the negative regulation of RhpP on the T3SS gene induction is obvious only when RhpC is absent, because deletion of the *rhpPC* locus did not significantly change the induction of *avrPto-luc* in the plant and MM. Thus, it is the coordination between *rhpP* and *rhpC* that plays a critical role in regulating the T3SS gene induction. It appears that RhpC, as a chaperone, keeps the function of RhpP under control in the cytoplasm by inhibiting the RhpP protease and facilitating its translocation to the periplasm. RhpP may play an important role in periplasmic protein quality control by degrading the functionally abnormal proteins of bacterium or imported peptides from environment. We conducted a preliminary analysis to compare the proteomes of the wild-type *Psph* and *ΔrhpC* strains, which revealed ~30 periplasmic proteins that were >1.5 fold more abundant in the *ΔrhpC* mutant (S1 Table). Most of these periplasmic proteins are ABC transporters involved in transportation of various small molecules. Whether any of these differentially expressed proteins are direct substrates of RhpP remains to be studied. Nonetheless, the presence of RhpP protein in the periplasm and cytoplasm in the wild-type *Psph* strain implies that the regulation of T3SS genes is coordinated with the periplasmic activities. RhpC plays a key role in protecting the T3SS regulation pathway and bacterial fitness from the detrimental effect of RhpP by directly inhibiting the RhpP protease activity in bacterial cytoplasm and targeting RhpP to the periplasm. The coordination of the two-component *rhpPC* module represents a novel mechanism underlying the fine regulation of T3SS genes in response to growth conditions.

## Materials and methods

### Plant materials and bacterial strains

*Arabidopsis att1* mutant plants [28] were used for the screening of *Psph* mutants with diminished induction of the *avrPto-luc* reporter. Bean (*Phaseolus valgaris* cv. Red Kidney) [52] was used for pathogenicity assays. Growth of plant materials was as described previously [21]. All *Psph* mutants were derived from the NPS3121 strain. *E*. *coli* DH5α was used for constructing all plasmids. *E*. *coli* BL21 was used for expression of recombinant proteins. NPS3121 and its derivatives were grown at 28°C in KB medium [53] containing appropriate antibiotics. *E*. *coli* strains were cultured in Luria-Bertani (LB) medium at 37°C. Antibiotics for selection of *Psph* strains were rifampicin, 25 mg/liter; kanamycin, 10 mg/liter; gentamicin,10 mg/liter; and spectinomycin, 50 mg/liter. Antibiotics for selection of *E*. *coli* were ampicillin, 100 mg/liter; kanamycin, 50 mg/liter; spectinomycin, 100 mg/liter; and gentamicin, 20 mg/liter.

### Construction of transposon-insertion library and screen of *rhpC*^-^ mutant

*Psph* NPS3121 strain carrying the pHM2::*avrPto-luc* reporter plasmid was used for construction of the EZ Tn*5*<KAN-2> (Epicentre, Madison, WI, U.S.A.) transposon insertion library, as described by Xiao et al. [21]. Briefly, electro-competent cells were mixed with transposon and transposase as instructed by the manufacturer. Following electroperation, the NPS3121 mutant library was plated on KB medium containing 10 mg of kanamycin per liter (selection for EZ::TN<KAN-2> transposon) and 10 mg of spectinomycin per liter (selection for pHM2::*avrPto-luc* plasmid).

To screen the mutants regulating *avrPto-luc* reporter in plant, the NPS3121 mutant colonies were grown overnight at 25°C in liquid KB medium containing spectinomycin and kanamycin. The bacteria were washed twice with sterile water, and then suspended in sterile water to optimal density at 600 nm (OD_600_) = 0.5 for inoculation. Six hour after injection into *Arabidopsis att1* plants, the inoculated leaves were excised and sprayed with 1 mM luciferin dissolved in 0.01% Tween-20, and the luciferase activity was determined using a cooled charge-couple device (CCD, Roper Scientific, Trenton, NJ, U.S.A.). A total of 6,000 colonies were screened. The transposon insertion sites were determined by a two stage semi-degenerated PCR, as described by Xiao et al [21]. To confirm the transposon insertion site in *rhpC*^-^ mutant, gene-specific primer *rhpC*-R and transposon-specific primer Kan2-SP3 were used to PCR amplify the transposon-flanking DNA. The PCR product was sequenced to determine the transposon insertion site.

### Analysis of *avrPto-luc* reporter activities in plant and MM

Bacteria containing the *avrPto-luc* reporter gene were grown in KB with appropriate antibiotics to OD_600_ = 2. The bacteria were washed twice with sterile water, and then resuspended in sterile water to OD_600_ = 0.5. To measure the reporter gene activity in plant, bacteria at OD_600_ = 0.5 were injected into *Arabidopsis att1* plants. The inoculated leaves were excised 6 h after injection and sprayed with 1 mM luciferin dissolved in 0.01% Tween-20, and the luciferase activity was determined using a cooled CCD (Roper Scientific, Trenton, NJ, U.S.A.). To measure the reporter gene activity in MM, bacteria were grown in KB with appropriate antibiotics overnight to OD_600_ = 2.0, washed three times with sterile water, resuspended in MM with appropriate antibiotics to OD_600_ = 0.1, and then cultured at 28°C with constant shaking for 6 h before the measurement. Bacterial culture (100 μl) was mixed with 1 μl of 1 mM luciferin in a 96-well plate, and the luciferase activities were determined using a cooled CCD (Roper Scientific, Trenton, NJ, U.S.A.). The luciferase activities of all measurements were normalized to the bacterial numbers. An *F* test (*P* < 0.05) was conducted to all quantitative experiments.

### Bacterial pathogenicity assays

Preparation of bacterial inoculum and bacterial growth assay were as previously described [21]. *Psph* NPS3121 wild type and mutant strains at 2×10^5^ CFU/ml were hand-injected into the primary leaves of 10-day-old bean plants. Bacterial growth were measured at 0, 2, and 4 days after inoculation. Disease symptoms on bean leaves were photographed 7 days after inoculation.

### Construction of plasmids

Oligo primers and plasmids used in this study are listed in S2 and S3 Tables, respectively. pBluescript-HA was modified from pBluescript-SK(+) in our previous study [21] and used for HA-tagging of genes at the 3′ end. Broad host plasmids pML122 [54] and pHM1 [55] with strong constitutive promoters were used to express genes in NPS3121 strain.

To construct the pML122::*rhpC-HA* plasmid, the *rhpC* gene from the NPS3121 strain was PCR-amplified using the RhpC-HA primer pair. The PCR product was digested with *Hind*III and *Nhe*I and cloned into the pBluescript-HA plasmid predigested with the same enzymes. After sequence confirmation, the pBluescript::*rhpC-HA* plasmid was digested by *Hind*III and *Bam*HI, and the insert was subsequently cloned into pML122 predigested with the same enzymes, resulting in pML122::*rhpC-HA*.

To construct the pHM1::*rhpP-FLAG* plasmid, the *rhpP* gene of the NPS3121 strain was PCR-amplified using the RhpP-FLAG (containing FLAG-tag DNA sequence) primer pair. The PCR product was digested with *Eco*RI and *Hind*III and cloned into the pGEM-T plasmid predigested with the same enzymes. After sequence confirmation, the pGEM-T::*rhpP-FLAG* plasmid was digested by *Eco*RI and *Hind*III, and the insert was subsequently cloned into pHM1 predigested with the same enzymes, resulting in pHM1::*rhpP-FLAG*.

To construct the pHM1::*hrpL-FLAG* plasmid, the *hrpL* gene from the NPS3121 strain was PCR-amplified using the HrpL-FLAG (containing FLAG tag DNA sequence) primers. The PCR product was digested with *Hind*III and *EcoR*I and cloned into the pGEM-T plasmid predigested with the same enzymes. After sequence confirmation, the pGEM-T::*hrpL-FLAG* plasmid was digested by *Hind*III and *Eco*RI, and the insert was subsequently cloned into pHM1 predigested with the same enzymes, resulting in pHM1::*hrpL-FLAG*.

To construct the pHM1::*hrpS-FLAG* plasmid, the *hrpS* gene from the NPS3121 strain was PCR-amplified using the HrpS-FLAG (containing FLAG tag DNA sequence) primers. The PCR product was digested with *EcoR*I and *Hind*III and cloned into the pGEM-T plasmid predigested with the same enzymes. After sequence confirmation, the pGEM-T::*HrpS-FLAG* plasmid was digested by *Eco*RI and *Hind*III, and the insert was subsequently cloned into pHM1 predigested with the same enzymes, resulting in pHM1::*hrpS-FLAG*.

To construct the pHM1::*hrpR-HA* plasmid, the *hrpR* gene from the NPS3121 strain was PCR-amplified using the HrpR-HA (containing HA-tag DNA sequence) primers. The PCR product was digested with *Pst*I and *Hind*III and cloned into the pGEM-T plasmid predigested with the same enzymes. After sequence confirmation, the pGEM-T::*hrpR-HA* plasmid was digested by *Pst*I and *Hind*III, and the insert was subsequently cloned into pHM1 predigested with the same enzymes, resulting in pHM1::*hrpR-HA*.

To construct the pHM1::*PSPPH_1783-FLAG*, pHM1::*AmiC-FLAG*, pHM1::*RpoA-FLAG* plasmid, the *PSPPH_1783*, *AmiC* (*PSPPH_5159*), *RpoA* (*PSPPH_4567*) were amplified from the NPS3121 strain by PCR and cloned into pHM1 using a ClonExpress II One Step Cloning Kit (Vazyme Biotech, Nanjing, China).

To construct the pGEX3X::*rhpC-GST* plasmid, the *rhpC* gene from the NPS3121 strain was PCR-amplified using the RhpC-GST primers. The PCR product was digested with *Bam*HI and *Eco*RI and cloned into the pGEM-T plasmid predigested with the same enzymes. After sequence confirmation, the pGEM-T::*rhpC* plasmid was digested by *Bam*HI and *Eco*RI, and the insert was subsequently cloned into pGEX3X predigested with the same enzymes, resulting in pGEX3X::*rhpC-GST*.

To construct the pMAL-p2X::*rhpP-MBP* plasmid, the *rhpP* gene from the NPS3121 strain was PCR-amplified using the RhpP-MBP primers. The PCR product was digested with *Eco*RI and *Bam*HI and cloned into the pGEM-T plasmid predigested with the same enzymes. After sequence confirmation, the pGEM-T::*rhpP-MBP* plasmid was digested by *Eco*RI and *Bam*HI, and the insert was subsequently cloned into pMAL-p2X predigested with the same enzymes, resulting in pMAL-p2X::*rhpP-MBP*.

### Construction of gene deletion mutants in *Pseudomonas* bacteria

To construct the *ΔrhpP* mutant, a 1.5-kb DNA fragment upstream of the *rhpP* open reading frame (ORF) was PCR-amplified using primers RhpP-FlankA (*Eco*RI and *Bam*HI). A 1-kb DNA fragment downstream of *rhpP* ORF was PCR-amplified using primers RhpP-FlankB (*Bam*HI and *Hind*III). The PCR products were digested with *Eco*RI and *Bam*HI and *Bam*HI and *Hind*III, respectively, and were ligated into pGEM-T, resulting in pGEM-T::*rhpPFlankA* and pGEM-T:: *rhpPFlankB*. After sequence confirmation, the pGEM-T::*rhpPFlankA* and pGEM-T::*rhpPFlankB* plasmids were digested by *Eco*RI and *Bam*HI and *Bam*HI and *Hind*III, and the inserts were subsequently cloned into pK18mobsacB [56], resulting in pK18mobsacB::*rhpPFlankAB*. This plasmid was then transformed into *E*. *coli* S17-1 which was used as a donor strain, and NPS3121 was used as a recipient strain. Both recipient strain and donor strain were cultured overnight, and then 50 μl of each were mixed and incubated for 2 days at 28°C. The bacteria were then collected and plated onto the KB plate containing rifampicin and kanamycin. After 2 days incubation, the rifampicin and kanamycin resistant colonies were single crossover merodiploid transconjugants. Pick several colonies and suspend the cells in a new tube and plate the suspension onto the KB/ rifampicin 10% sucrose plate. The sucrose rifampicin resistant colonies were regarded as the deletion mutant and further verified by PCR and sequencing.

To construct the *ΔrhpC* mutant, a 1.5-kb DNA fragment upstream of *rhpC* ORF was PCR-amplified using primers RhpC-FlankA (*Eco*RI and *Bam*HI). A 1-kb DNA fragment downstream of *rhpC* ORF was PCR-amplified using primers RhpC-FlankB (*Bam*HI and *Hind*III). The PCR products were digested with *Eco*RI and *Bam*HI and *Bam*HI and *Hind*III, respectively, and were ligated into pGEM-T, resulting in pGEM-T:: *rhpCFlankA* and pGEM-T::*rhpCFlankB*. After sequence confirmation, the pGEM-T::*rhpCFlankA* and pGEM-T::*rhpCFlankB* plasmids were digested by *Eco*RI and *Bam*HI and *Bam*HI and *Hind*III, and the inserts were subsequently cloned into pK18mobsacB, resulting in pK18mobsacB::*rhpCFlankAB*. The remaining procedures were the same as described for construction of the *ΔrhpP* mutant.

To construct the *ΔrhpPC* mutant, a 1.5-kb DNA fragment upstream of *rhpP* ORF was PCR-amplified using primers RhpP-FlankA (*Eco*RI and *Bam*HI). A 1-kb DNA fragment downstream of *rhpC* ORF was PCR-amplified using primers RhpC-FlankB (*Bam*HI and *Hind*III). The PCR products were cloned into pK18mobsacB, resulting in pK18mobsacB::*rhpCFlankAB*. The remaining procedures were the same as described for construction of the *ΔrhpP* mutant.

The same procedures described above were also used for construction of the *ΔgspD and ΔgspE* deletion mutants, except different primers were used to amplify DNA flanking the corresponding genes. Primers GspD-FlankA and GspD-FlankB were used for construction of *ΔgspD*, and primers GspE-FlankA and GspE-FlankB were used for construction of *ΔgspE*.

### Site-directed mutagenesis of *rhpP*

To generate RhpP^H279D^, RhpP^H279T^, and RhpP^H176A,E177A,H180A^ mutants, primer sets (RhpP^H279D^, RhpP^H279T^, and RhpP^H176A,E177A,H180A^) containing corresponding mutations and the pGEM-T::*rhpP-FLAG* construct as template were used in PCR with the high fidelity DNA polymerase Phusion (Thermo Scientific, Waltham, MA, U.S.A.). The PCR product was digested with DMT enzyme (Transgen Biotech, Beijing, China) and then transformed into *E*. *coli*. Mutations in the resulting clones were verified by sequencing.

### RNA extraction and real-time PCR analysis of gene expression

Bacterial RNA was extracted using a modified hot phenol method [57]. RQ1 DNase (Promega, Madison, WI, U.S.A.) treatment was used to remove the contaminating DNA in RNA samples. Real-time PCR assay of gene expression was performed as described [58]. Briefly total RNA (10 μg) was reverse-transcribed using random primers. The reactions were first treated with RNase and then used as template for real-time PCR analyses with primer pairs rhpP-5'/-3 and rhpC-5'/-3 for *rhpP* and *rhpC* respectively, and primer pairs 16S rRNA, *recA*, and *rpoD* as internal controls. The relative expression levels of *rhpP* and *rhpC* were normalized to 16S rRNA, *rpoD* and *recA*.

### Protein pull-down assay

*E*. *coli* BL21 strains containing pGEX-3X-*rhpC* and pGEX-3X plasmids were cultured in LB at 37°C to OD_600_ = 0.5 before adding 1 mM IPTG. The bacteria were then cultured overnight at 16°C to induce the production of the recombinant proteins. RhpC-GST and GST were purified using the Glutathione Sepharose ^™^ 4B (GE Healthcare Bio-Science AB, Uppsala, Sweden), following the manufacture’s instruction.

The wild type *Psph* strains containing the pHM1::*rhpP-FLAG*, pHM1::*rhpP*^*H279D*^*-FLAG*, pHM1::*rhpP*^*H279T*^*-FLAG*, and pHM1::*phpP*^*H176A*,*E177A*,*H180A*^*- FLAG* were used to produce the corresponding RhpP and mutant proteins. The bacteria were cultured in 200 ml KB/rifampicin and spectinomycin at 28°C with constant shaking at 220 rpm. The bacteria were washed once with ice-cold PBS (10 mM Tris-HCl pH8.0, 1 mM EDTA pH 8.0, 150 mM NaCl), sonicated in ice-cold PBS, and then centrifuged at 4°C and 10,000 x *g* for 30 min. The supernatant was mixed with 1/1000 PBS-pre-washed anti-FLAG M2 Affinity Gel (Sigma Aldrich, St. Louis, MO, U.S.A.) and incubated at 4°C for 2 h with constant shaking. RhpP-FLAG protein bound to the beads was centrifuged, washed three times with PBS, and the protein was then washed off the beads using 3×FLAG peptide (200 μg/ml). The purified protein was examined using Western blot.

RhpC-GST or GST bound to the Glutathione Sepharose beads was mixed with the purified RhpP-FLAG protein and incubated at 4°C for 2 h with constant shaking. Aliquot a small volume as input. The remaining of the mixture was centrifuged at 50 x *g* for 5 min, the precipitate was then washed three times (each time for 5 min) with ice-cold PBS, and then three times (each time for 5 min) with ice-cold wash solution (10 mM Tris-HCl pH8.0, 1 mM EDTA pH 8.0, and 500 mM NaCl). Protein bound to the beads were eluted with elution buffer (20 mM reduced glutathione, 50 mM Tris-HCL pH 8.0) and analyzed with Western blot as previously described [21].

### Protein localization assay

The procedures described previously [59, 60] were applied for periplasmic and cytoplasmic protein assay. Bacteria cultured in KB or MM were centrifuged and then suspended in ice-cold osmotic solution II (OS II: 20 mM Tris-HCl, pH8.0; 2.5 mM EDTA; 2 mM CaCl_2_) to OD_600_ = 3.0. Aliquot 10 μl of the bacterial suspension and slowly add in 1 ml ice-cold osmotic solution I (OS I: 20 mM Tris-HCl, pH8.0; 2.5 mM EDTA; 2 mM CaCl2; 20% (w/v) sucrose). After 10 min of incubation in ice, the bacteria were centrifuged and then suspended in 1 ml ice-cold OS II, and continued incubation in ice for 20 min. Periplasmic contents defused into the solution during the incubation was collected by centrifugation. The bacterial pellet was suspended in 1 ml OS II. Cytoplasmic protein were released into solution by sonication and collected by ultracentrifugation.

To test protein secretion into the culture medium, bacteria were first cultured in 10 ml KB medium to OD_600_ = 2, and then transferred into 500 ml KB for continuing culture to OD_600_ = 1. For secretion assay in KB medium, the bacteria were separated from the KB medium by centrifugation. For secretion assay in MM, the 500 ml KB culture were centrifuged, and the bacteria were washed three times with MM and then suspended in 500 ml MM. After 6 h culture in MM, the bacteria were separated from the MM by centrifugation. The medium was filtrated with the 0.45 μM membrane to remove the bacterial residuals, and then mixed with equal volume of ice-cold 20% TCA. After 30 min incubation in ice, the mixture was centrifuged at 10,000 x *g* for 15 mins, and the pellet was washed three times with ice-cold acetone, and then boiled in SDS sample buffer for Western blot analysis.

### In vitro digestion of HrpL protein by RhpP

RhpP-FLAG and RhpP^H279T^-FLAG proteins were produced by pHM1::*rhpP-FLAG* and pHM1::*rhpP*^*H279T*^*-FLAG* plasmids, respectively, in *Psph* strain. HrpL-FLAG protein was produced by the pHM1::*hrpL-FLAG* plasmid in *E*. *coli* BL21 strain. The proteins were purified by the anti-FLAG M2 Affinity Gel and eluted using 3×FLAG peptide (200 μg/ml). RhpC-GST and GST were purified using glutathione Sepharose beads. The purified HrpL-FLAG protein was adjusted to a concentration of 200μg/ml in PBS buffer, and all the other purified were adjusted to a concentration of 400μg/ml in PBS buffer. For each reaction, 50 μl of purified HrpL-FLAG protein was mixed with equal volume of PBS buffer or other purified proteins. EDTA was added to a final concentration of 5mM. Aliquot of 50 μl was taken from each reaction and mixed with SDS sample buffer as 0 h control, and the remaining volumes were incubated at 28°C for 3 h before examined by Western blot analysis.

### Western blot analysis

SDS-PAGE and Western blot analysis was conducted according to the procedures described previously [21]. Anti-FLAG antibody and anti-HA antibody were purchased from Sigma Aldrich (St Louis, MO, USA) and Abmart (Shanghai, China), respectively.

### Sequence alignment and phylogenetic analysis of RhpC and RhpP

Homologous sequences of RhpC and RhpP were downloaded from EnsemblBacteria [61] (http://bacteria.ensembl.org/index.html) and the *Pseudomonas* Genome [62] (http://pseudomonas.com/) databases with RhpC and RhpP from *P*. *s*. pv. *phaseolicola* 1448A as queries. All the sequences were aligned with ClustalW, and the UPGMA phylogenetic tree were then constructed with MEGA5 using default parameters [63].

### Prediction of isoelectric point and secondary structure of RhpC protein

The isoeletric point of RhpC protein was calculated with 15 methods included in Isoelectric Point Calculator (IPC, http://isoelectric.org/calculate.php). The secondary structure of RhpC was predicted with PredictProtein (https://predictprotein.org/), the most widely used server for structure prediction.

### Signal peptide and Tat-targeting peptide prediction

SignalP [64] (http://www.cbs.dtu.dk/services/SignalP/) and TatP [65] (http://www.cbs.dtu.dk/services/TatP/) were used to predict the signal peptide and possible Tat-targeting peptide in RhpP proteins based on the classifications of bacteria.

### Subcellular location prediction

Two widely used online servers, CELLO [31, 32] (http://cello.life.nctu.edu.tw/) and PredictProtein (https://predictprotein.org/), were used to predict the subcellular location of RhpC.

## Supporting information

S1 Table Up-regulated periplasmic proteins in the *ΔrhpC* mutant identified by proteomic analysis. (DOCX)

S2 Table Primers used in this study. (DOCX)

S3 Table Plasmid used in this study. (DOCX)

S1 Fig Regulatory network for the *Pseudomonas* T3SS genes. The role of each regulator was described in the Introduction. The functions of RhpC and RhpP were described by this study. Solid lines indicate known working mechanisms. Dashed lines indicate working mechanisms unknown.
(TIF)

S2 Fig Bacterial numbers of the wild type *Psph* and *ΔrhpC* after inoculation in the wild type Arabidopsis Col-0 and *att1* mutant. Bacteria were inoculated at 10^6^ cfu/ml. Bacterial numbers were measured at 0, 2, and 4 days after inoculation.
(TIF)

S3 Fig Phylogenetic analysis and alignment of RhpC and its homologous proteins. (A) Phylogenetic analysis. (B) Alignment of RhpC and its homologous proteins. The alignment order in (B) is the same as (A) from left to right. The conserved amino acid residues are shadowed.
(TIF)

S4 Fig Prediction of isoelectric point and secondary structure of RhpC protein with multiple methods. (A) Prediction of isoelectric point. (B) Prediction of secondary structure of RhpC protein. H, α-helix; E, β-sheet.
(TIF)

S5 Fig Phylogenetic analysis of RhpP homologues in *Pseudomonas*. Strains indicated by the filled circles carry the *rhpPC* locus. Strains indicated by the open circles carry the *rhpP* gene only.
(TIF)

S6 Fig Phylogenetic analysis of RhpP homologous proteins in different bacteria. Strains indicated by the filled circles carry the *rhpPC* locus. Strains without any marks carry the *rhpP* gene only.
(TIF)

S7 Fig Alignment of RhpP with the out-secreted M4 metalloproteases. Red arrowheads are predicted zinc binding sites. Blue arrowheads are predicted active sites. Conserved M4 neutral protease domain is marked with black line.
(TIF)

S8 Fig Alignment of RhpP with RhpP homologues that are coupled by RhpC homologues. Red arrowheads are predicted zinc binding sites. Blue arrowheads are predicted active sites. Conserved M4 neutral protease domain is marked with black lines.
(TIF)

S9 Fig Primers for PCR, RT-PCR, and real time PCR analyses of the *rhpPC* locus gene expression. Primer pairs for different PCR analyses are coded with different colors. The sequences for the PCR primers are listed in Table S1.
(TIF)

S10 Fig Growth curves of *ΔrhpPC* mutant strains carrying the empty pHM1 vector or pHM1 expressing RhpP-FLAG, RhpP^H279D^- FLAG, and RhpP^H279T^- FLAG proteins in KB. (TIF)

S11 Fig SignalP prediction of the Sec signal peptide in RhpP. (A) Elastase of *Pseudomonas aeruginosa* to show predication of a typical signal peptide. (B) SignalP prediction of RhpP from *Psph*.
(TIF)

S12 Fig TatP predication of Tat signal peptide in RhpP. (TIF)

S13 Fig Alignment of RhpC with TatB from *P*. *aeruginosa*, *P*. *s*. pv. *tomato* DC3000, and *P*. *s*. pv. *phaseolicola* 1448A strains. (TIF)
