# Supplementary material for: Two components of the rhpPC operon coordinately regulate the type III secretion system and bacterial fitness in Pseudomonas savastanoi pv. phaseolicola
Source: PLoS Pathog. 2019 Apr 18;15(4):e1007673. doi: 10.1371/journal.ppat.1007673 (PMC6490944; doi:10.1371/journal.ppat.1007673)
Supplement: S1 Table — (DOCX) [file ppat.1007673.s001.docx]

| **S1 Table. Up-regulated periplasmic proteins in the** *Δ****rhpC* mutant identified by proteomic analysis.** | |  |
| --- | --- | --- |
| **Accession** | **Description** | *Δ****rhpC*/WT** |
| **KB** |  |  |
| AAZ34542.1 | periplasmic glucan biosynthesis protein | 0.812 |
| AAZ34742.1 | ferric siderophore transporter, periplasmic energy transduction protein TonB | 0.870 |
| AAZ33878.1 | periplasmic glucans biosynthesis protein MdoG | 0.910 |
| AAZ37251.1 | periplasmic substrate-binding protein | 1.176 |
| AAZ35794.1 | L-arabinose ABC transporter, periplasmic L-arabinose-binding protein | 1.203 |
| AAZ33396.1 | putrescine ABC transporter, periplasmic putrescine-binding protein | 1.22 |
| AAZ35822.1 | glycine betaine/L-proline ABC transporter, periplasmic substrate-binding protein | 1.228 |
| AAZ35285.1 | Predicted periplasmic lipoprotein involved in iron transport | 1.252 |
| AAZ33809.1 | phosphate ABC transporter, periplasmic phosphate-binding protein, putative | 1.264 |
| AAZ33190.1 | polyamine ABC transporter, periplasmic polyamine-binding protein | 1.386 |
| AAZ35352.1 | putrescine ABC transporter, periplasmic putrescine-binding protein | 1.538 |
| AAZ33143.1 | iron compound ABC transporter, periplasmic iron compound-binding protein | 1.657 |
| AAZ35146.1 | putrescine ABC transporter, periplasmic putrescine-binding protein | 1.699 |
| AAZ37684.1 | ABC transporter, periplasmic substrate-binding protein | 1.718 |
| AAZ34925.1 | zinc ABC transporter, periplasmic zinc-binding protein | 1.777 |
| AAZ37108.1 | amino acid ABC transporter, periplasmic amino acid-binding protein | 1.815 |
| AAZ34045.1 | histidine transporter, periplasmic histidine-binding protein | 1.838 |
| AAZ35451.1 | iron ABC transporter, periplasmic iron-binding protein | 1.852 |
| AAZ35696.1 | amino acid ABC transporter, periplasmic amino acid-binding protein | 1.956 |
| AAZ34867.1 | amino acid ABC transporter, periplasmic amino acid-binding protein | 2.139 |
| AAZ36153.1 | ribose ABC transporter, periplasmic ribose-binding protein | 2.175 |
| **MM** |  |  |
| AAZ36480.1 | sulfate ABC transporter, periplasmic sulfate-binding protein | 0.756 |
| AAZ33002.1 | glucose ABC transporter, periplasmic glucose-binding protein, putative | 0.793 |
| AAZ36730.1 | sugar ABC transporter, periplasmic sugar-binding protein | 0.812 |
| AAZ37344.1 | sugar ABC transporter, periplasmic sugar-binding protein | 0.818 |
| AAZ37918.1 | mannitol ABC transporter, periplasmic mannitol-binding protein | 0.824 |
| AAZ34529.1 | sugar ABC transporter, periplasmic sugar-binding protein | 0.843 |
| AAZ33267.1 | peptide ABC transporter, periplasmic peptide-binding protein | 0.868 |
| AAZ33032.1 | polyamine ABC transporter, periplasmic polyamine-binding protein | 0.872 |
| AAZ37684.1 | ABC transporter, periplasmic substrate-binding protein | 1.177 |

Note: The numbers in *ΔrhpC*/WT are ln2 transformed.
