# Supplementary material for: Two components of the rhpPC operon coordinately regulate the type III secretion system and bacterial fitness in Pseudomonas savastanoi pv. phaseolicola
Source: PLoS Pathog. 2019 Apr 18;15(4):e1007673. doi: 10.1371/journal.ppat.1007673 (PMC6490944; doi:10.1371/journal.ppat.1007673)
Supplement: S2 Table — (DOCX) [file ppat.1007673.s002.docx]

**S2 Table. Primers used in this study.**

| **Primer** | **Forward (5′to3′)** | **Reverse (5′to3′)** | **Purpose** |
| --- | --- | --- | --- |
| RhpC/Kan2-SP3 | ATGTACGACGACCGTAACCCCCT | GCAATGTAACATCAGAGATTTTGAG | Confirmation of transposon insertion in *rhpC-* mutant |
| rhpP-F/rhpC-R  rhpP-F/R  rhpC-F/R  rhpP-5'/-3'  rhpC-5'/-3'  16S rRNA  recA  rpoD  rhpC-RT | ATGTACGACGACCGTAACCCCC  ATGTACGACGACCGTAACCCCC  ATGAAAGTCTGTCTAGTGCAATCCGG  CTGAGCAACGCCTACTGGAA  AGTCGATACGTCCACCCTTG  GTAGTGGGGGATAACGCTCG  ATCACGCCGATTTTCATGCG  GTTCGGCAAGTGCAACCATT  CTAGAGCTTGCCGGGCTTTACG | CTAGAGCTTGCCGGGCTTTACG  CTAAACCTTGACCCCGACTTCCTTC  CTAGAGCTTGCCGGGCTTTACG  CCGAAGACATCGGCGAAATG  TGTTCGTCAAACGTGAGGCA  GCCTTGGTGAGCCATTACCT  AGGCAGAAATCGAAGGCGAA  AAGACGAAGTCGAAAGCGGT | For PCR and RT-PCR of *rhpPC*  For PCR and RT-PCR of *rhpP*  For PCR and RT-PCR of *rhpC*  For real time PCR of *rhpP* expression  For real time PCR of *rhpC* expression  For real time PCR of 16S *rRNA*  For real time PCR of *recA*  For real time PCR of *rpoD*  For *rhpPC* reverse-transcription |
| RhpP- FlankA | ACGAATTCCAGGAGCCGCTTTCAAAAAACTCCAG | TCGGATCCCCGAACACAAAGGTGCTTGAAATGAAAG | DNA fragment upstream of *rhpP,* for deletion of *rhpP* and *rhpPC* |
| RhpP- FlankB | GGGGATCCGATGAACTCCTGTTCTAATGCACCAACG | CCAAGCTTGCCGATCAAATCAAGGATCAGGCATTC | DNA fragment downstream of *rhpP,* for deletion of *rhpP* |
| RphC-FlankA | ACGAATTCGTCGCAGCGCGGGCAGCGCGTA | AAGGATCCGACAGGCCTGTTTGAAAACAATACGGCG | DNA fragment upstream of *rhpC,* for deletion of *rhpC* |
| RphC-FlankB | TCGGATCCTTCAAGCACCTTTGTGTTCGGCTAAACC | CCAAGCTTCATCTGAATGAACCACTGTGGCGTGG | DNA fragment downstream of *rhpC* for deletion of *rhpC* and *rhpPC* |
| GspD-FlankA | GAATTCTCCAGCTGATCCGCAACGTC | GGATCCAGCGGTGTCAGTTTCTGCCG | DNA fragment upstream of *gspD* for deletion of *gspD* |
| GspD-FlankB | GGATCCGGGTGTGCGCAAGGTAGCGAT | AAGCTTGACCAACACGTTAGGCGCGG | DNA fragment downstream of *gspD* for deletion of *gspD* |
| GspE-FlankA | GGATCCATGCCCCAGATCAGCGCGATCA | CTGCAGGTTTATTCAAGTATCGCGCGCTGGA | DNA fragment upstream of *gspE* for deletion of *gspE* |
| GspE-FlankB | CTGCAGGTTACAGCACTCTCCGCATGGAGA | AAGCTTTGCGGAACGCCGTAGTTGGTCAGC | DNA fragment downstream of *gspE* for deletion of *gspE* |
| RhpC-HA | TTAAGCTTATGAAAGTCTGTCTAGTGCAATCCGGCG | CTAGCTAGCGAGCTTGCCGGGCTTTACGCGTTG | For pML122::*rhpC-HA* construct |
| RhpC-HA-F/R | AAGCTTTATGAAAGTCTGTCTAGTGCAATCCGGCG | GAATTCTTAAGCGTAGTCTGGGACGTCGTATGGGTAGAGCTTGCCGGGCTTTACGCGTTG | For pHM1::*rhpC-HA* construct |
| RhpP-FLAG | AAGCTTTATGTACGACGACCGTAACCCCCT | GAATTCTTACTTATCGTCGTCATCCTTGTAATCAACCTTGACCCCGACTTCCTTCC | For pHM1:*:rhpP-FLAG* construct |
| RhpC-GST | GGATCCCCATGAAAGTCTGTCTAGTGCAATC | GAATTCCTAGAGCTTGCCGGGCTTTACG | For pGEX3X::*rhpC* construct |
| RhpP-MBP | GAATTCATGTACGACGACCGTAACCCCC | GGATCCCTAAACCTTGACCCCGACTTCC | For pMAL-p2X::*rhpP* construct |
| HrpL-FLAG | AAGCTTTATGTTTCCGAACCTAGTGATCCTTGATGC | GAATTCTTACTTATCGTCGTCATCCTTGTAATCGGCGAACGGGTCAATCTGCTGCT | For pHM1::*hrpL-FLAG* construct |
| HrpS-FLAG | AAGCTTTATGGATCTTGATGAGGGGTTTGATGACGAC | GAATTCTTACTTATCGTCGTCATCCTTGTAATCGATCTGCAACTCCTTGATACGTC | For pHM1::*hrpS-FLAG* construct |
| HrpR-HA  RpoA-FLAG (PSPPH_4567)  PSPPH_1783-FLAG | AAGCTTTATGAGCACAGACATTGATAAGGGCGTCCG  GACCATGATTACGCCAAGCTTTATGCAGATTTCGGTAAATG  GACCATGATTACGCCAAGCTTTATGAGCGACAGACCATACG | CTGCAGTTAAGCGTAGTCTGGGACGTCGTATGGGTATGCAGCAACTCCCAACTCCTTCAT  GTAAAACGACGGCCAGTGAATTCTTACTTATCGTCGTCATCCTTGTAATCGGCCGTAGCCTTGTCGTCCT  GTAAAACGACGGCCAGTGAATTCTTACTTATCGTCGTCATCCTTGTAATCGGCAGGAAAGTGCAATGCG | For pHM1::*hrpR-HA* construct  For pHM1::*RpoA-FLAG* construct  For pHM1::*PSPPH_1783-FLAG* construct |
| AmiC-FLAG (PSPPH_5159) | TGACCATGATTACGCCAAGCTTTTTGAGATGTTCCATGCACC | GTAAAACGACGGCCAGTGAATTCTTACTTATCGTCGTCATCCTTGTAATCGACCTGCTGTTCCTGCTTCT | For pHM1:: *AmiC -FLAG* construct |
| RhpP^H279D^ | GACCGCGGCGGCGTAGACATCAATTC | CTACGCCGCCGCGGTCCTTGGTGCCA | Site-directed mutagenesis of *rhpP* |
| RhpP ^H279T^ | GACCGCGGCGGCGTATACATCAATTC | ATACGCCGCCGCGGTCCTTGGTGCCA | Site-directed mutagenesis of *rhpP* |
| RhpP^H176A, E177A, H180A^ | GAAACTCTGCACCCCGGCGGTCAGGGCGGCGCCGATTACT | GCCGCCCTGACCGCCGGGGTGCAGAGTTTCACCTCGAACC | Site-directed mutagenesis of *rhpP* |
