# Supplementary material for: Two components of the rhpPC operon coordinately regulate the type III secretion system and bacterial fitness in Pseudomonas savastanoi pv. phaseolicola
Source: PLoS Pathog. 2019 Apr 18;15(4):e1007673. doi: 10.1371/journal.ppat.1007673 (PMC6490944; doi:10.1371/journal.ppat.1007673)
Supplement: S3 Table — (DOCX) [file ppat.1007673.s003.docx]

**S3 Table. Plasmid used in this study.**

| **Plasmids** | **Description** | **Reference** |
| --- | --- | --- |
| pGem7Z | Cloning and sequencing | Promega, Madison, WI,U.S.A. |
| pGEM-T | Cloning and sequencing | Promega, Madison, WI, U.S.A. |
| pK18mobsacB | Suicide plasmid | [55] |
| pBluescript-HA | Modified from pBluescript-SK(+), for HA-tagging and sequencing | [21] |
| pHM1 | Broad-host plasmid | [54] |
| pGEX-3X | Vector for expression of GST fusion protein | GE Healthcare, Uppsala, Sweden |
| pHM2 | Modified from pHM1, for *avrPto-luc* reporter expression | [21] |
| pML122 | Broad-host plasmid | [55] |
| pML122::*rhpC-HA* | pML122 plasmid for expression of RhpC-HA | This study |
| pHM1::*rhpC-HA* | pHM1 plasmid for expression of RhpC-HA | This study |
| pHM2::*avrPto-luc* | *avrPto-luc* reporter in pHM2 | [21] |
| pHM1::*rhpP-FLAG* | *RhpP* under lacZ promoter with FLAG-tagged at C-terminus | This study |
| pHM1::*rhpP^H279D^-FLAG* | Derived from pHM1::*rhpP-FLAG*, with His_279_ replaced by Ala | This study |
| pHM1::*rhpP^H279T-^FLAG* | Derived from pHM1::*rhpP-FLAG*, with His_279_ replaced by Thr | This study |
| pHM1:: *rhpP^H176A, E177A ,H180A^-FLAG* | Derived from pHM1:: *rhpP-FLAG*, with H_176_, E_177_ and H_180_ replaced by Ala | This study |
| pHM1::*hrpL-FLAG* | *hrpL* under *lacZ* promoter with FLAG-tagged at C-terminus | This study |
| pHM1::*hrpS-FLAG* | *hrpS* under *lacZ* promoter with FLAG-tagged at C-terminus | This study |
| pHM1::*hrpR-HA* | *hrpR* under *lacZ* promoter with HA-tagged at C-terminus | This study |
| pHM1::*RpoA-FLAG* | *RpoA* under *lacZ* promoter with FLAG-tagged at C-terminus | This study |
| pHM1::*PSPPH_1783-FLAG* | *PSPPH_1783* under *lacZ* promoter with FLAG-tagged at C-terminus | This study |
| pHM1:: *AmiC -FLAG* | *PSPPH_5159* under *lacZ* promoter with FLAG-tagged at C-terminus | This study |
| pGEX3X::*rhpC* | *rhpC* under *lacZ* promoter with GST-tagged at N-terminus | This study |
| pMAL-p2X::*rhpP* | *rhpP* under *lacZ* promoter with MBP-tagged at N-terminus | This study |
| pK18mobsacB:: *rhpP-FlankAB* | For *rhpP* deletion | This study |
| pK18mobsacB:: *rhpC-FlankAB* | For *rhpC* deletion | This study |
| pK18mobsacB:: *rhpPC-FlankAB* | For *rhpPC* operon deletion | This study |
| pK18mobsacB:: *gspD-FlankAB* | For *gspD* deletion | This study |
| pK18mobsacB:: *gspE-FlankAB* | For *gspE* deletion | This study |
