# Supplementary figures and images for: Two components of the rhpPC operon coordinately regulate the type III secretion system and bacterial fitness in Pseudomonas savastanoi pv. phaseolicola
Source: PLoS Pathog. 2019 Apr 18;15(4):e1007673. doi: 10.1371/journal.ppat.1007673 (PMC6490944; doi:10.1371/journal.ppat.1007673)

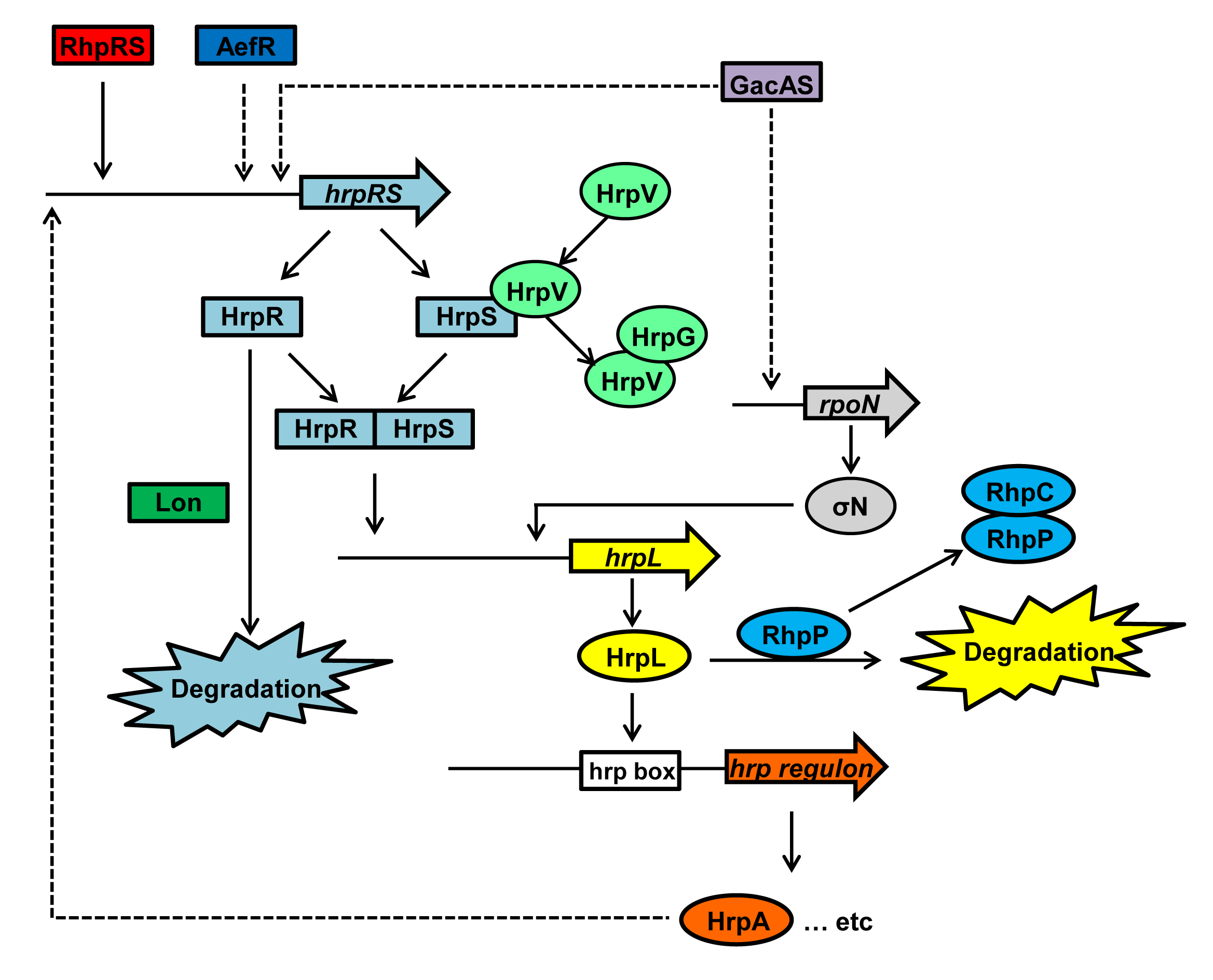

Supplement: S1 Fig — The role of each regulator was described in the Introduction. The functions of RhpC and RhpP were described by this study. Solid lines indicate known working mechanisms. Dashed lines indicate working mechanisms unknown. (TIF) [file ppat.1007673.s004.tif]

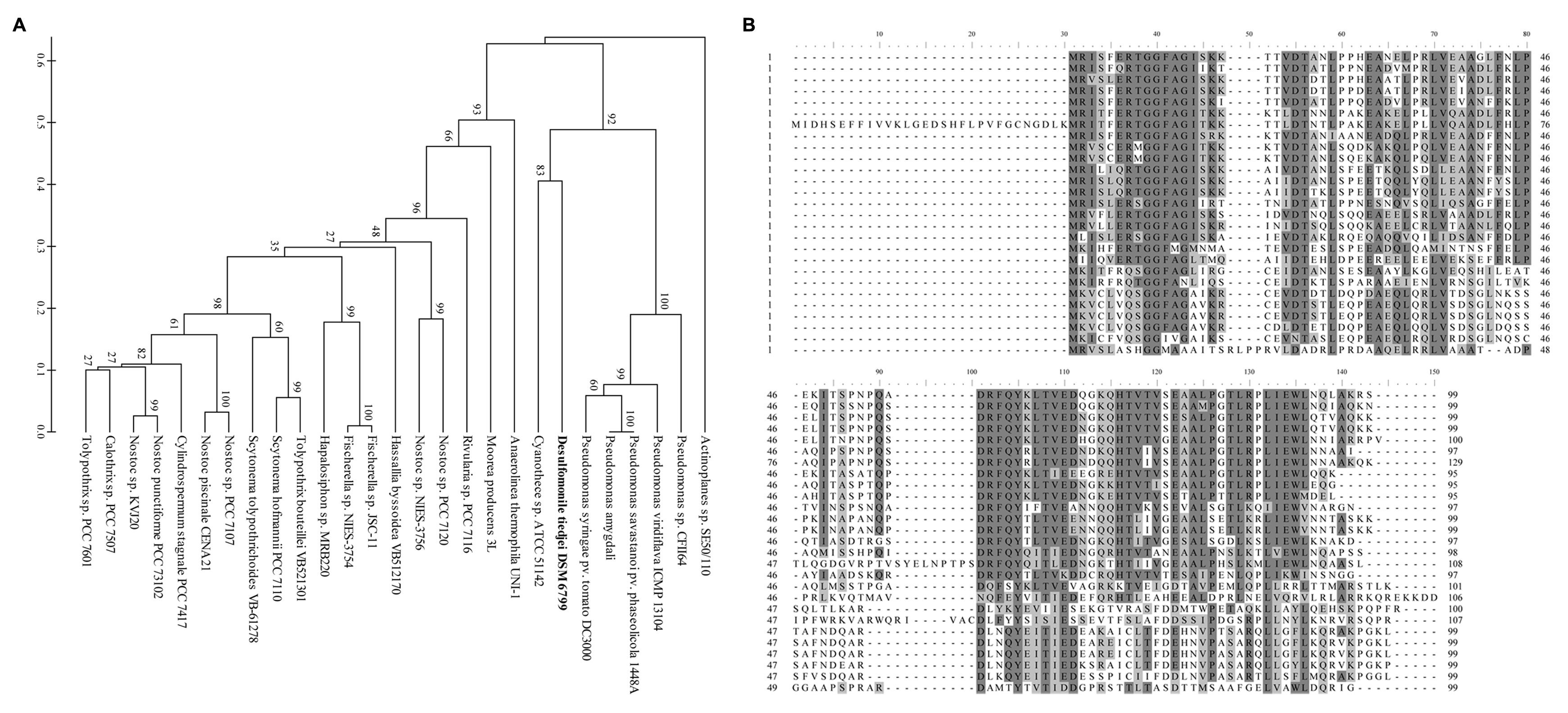

Supplement: S3 Fig — (A) Phylogenetic analysis. (B) Alignment of RhpC and its homologous proteins. The alignment order in (B) is the same as (A) from left to right. The conserved amino acid residues are shadowed. (TIF) [file ppat.1007673.s006.tif]

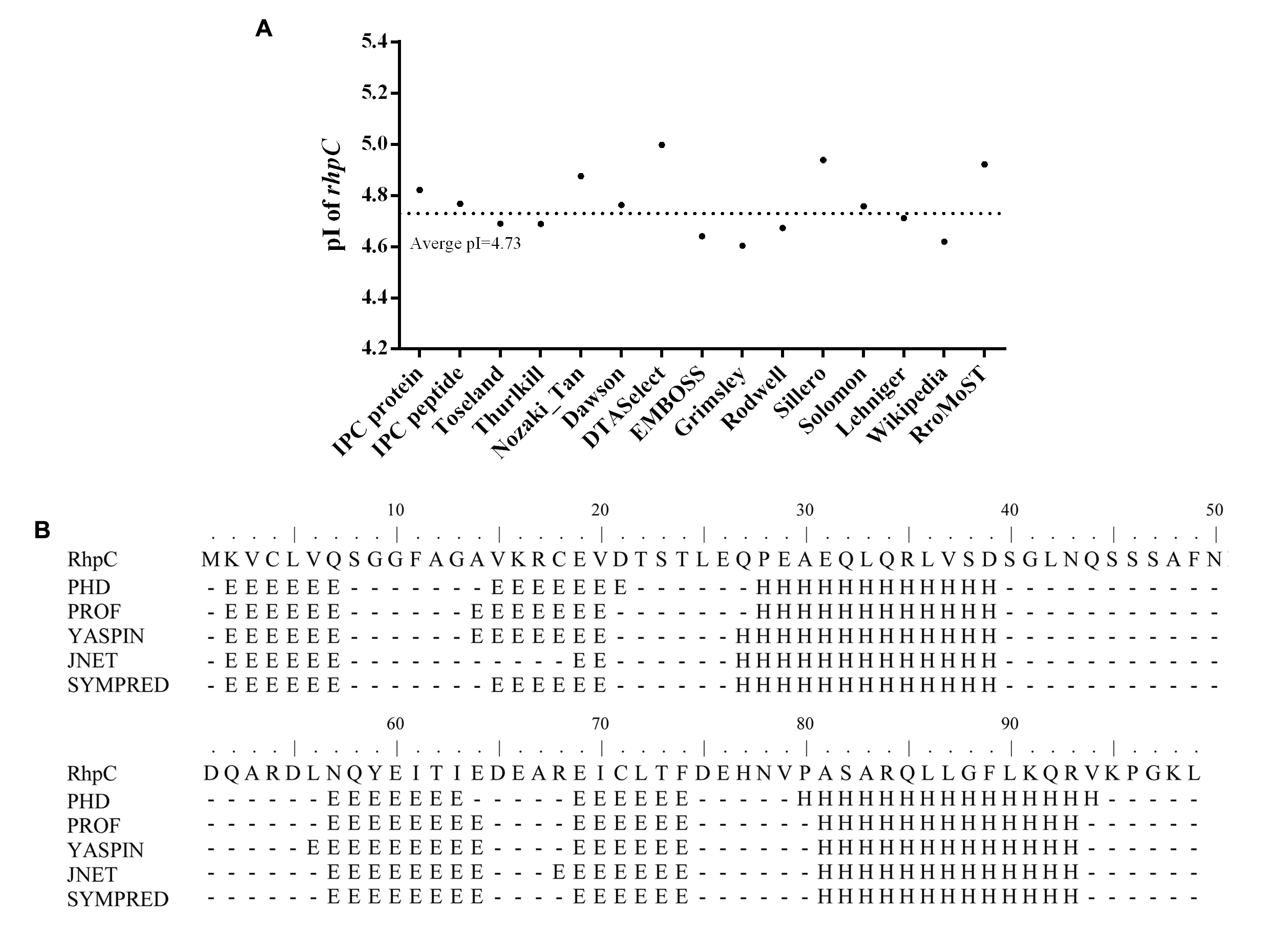

Supplement: S4 Fig — (A) Prediction of isoelectric point. (B) Prediction of secondary structure of RhpC protein. H, α-helix; E, β-sheet. (TIF) [file ppat.1007673.s007.tif]

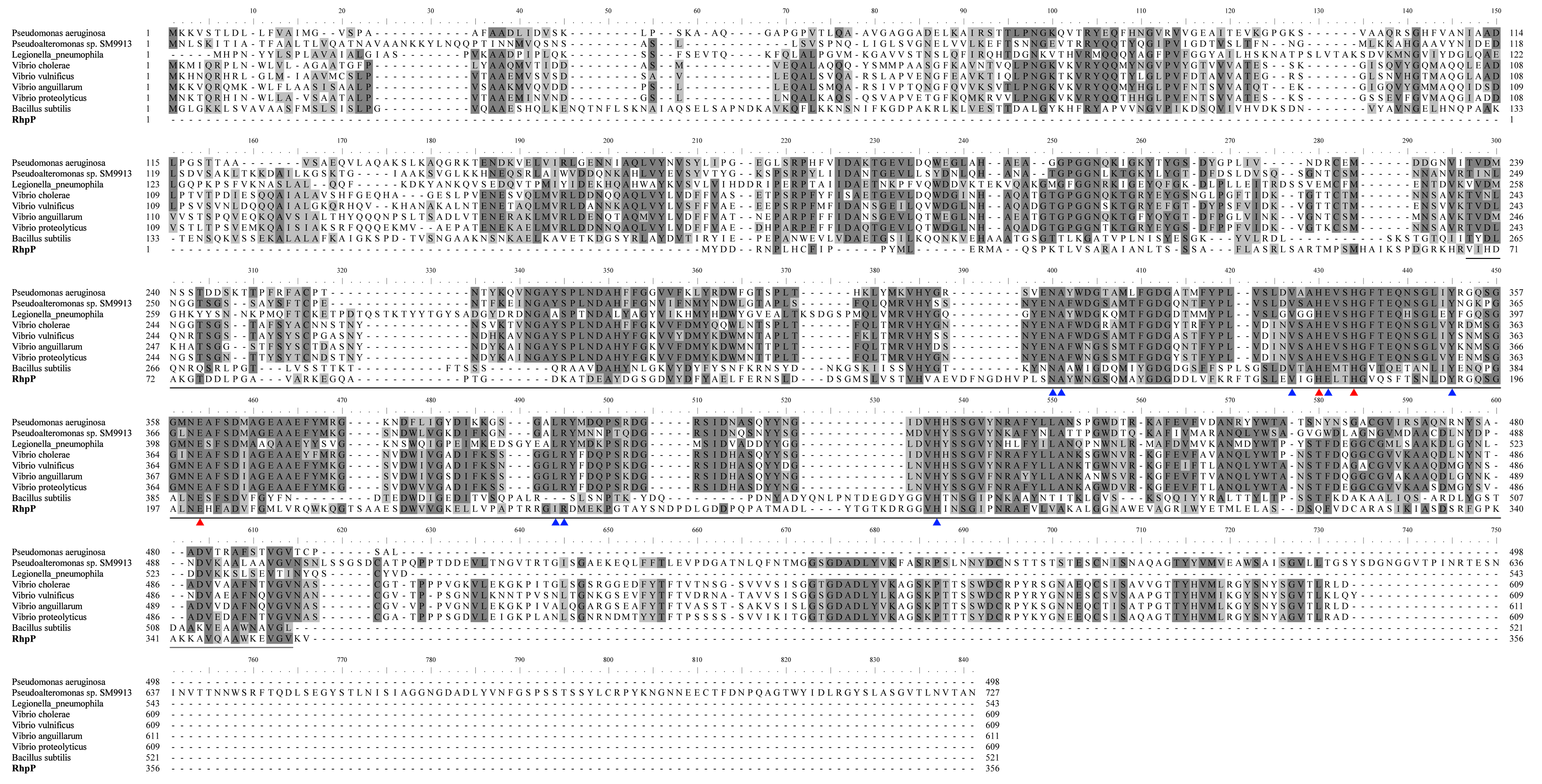

Supplement: S7 Fig — Red arrowheads are predicted zinc binding sites. Blue arrowheads are predicted active sites. Conserved M4 neutral protease domain is marked with black line. (TIF) [file ppat.1007673.s010.tif]

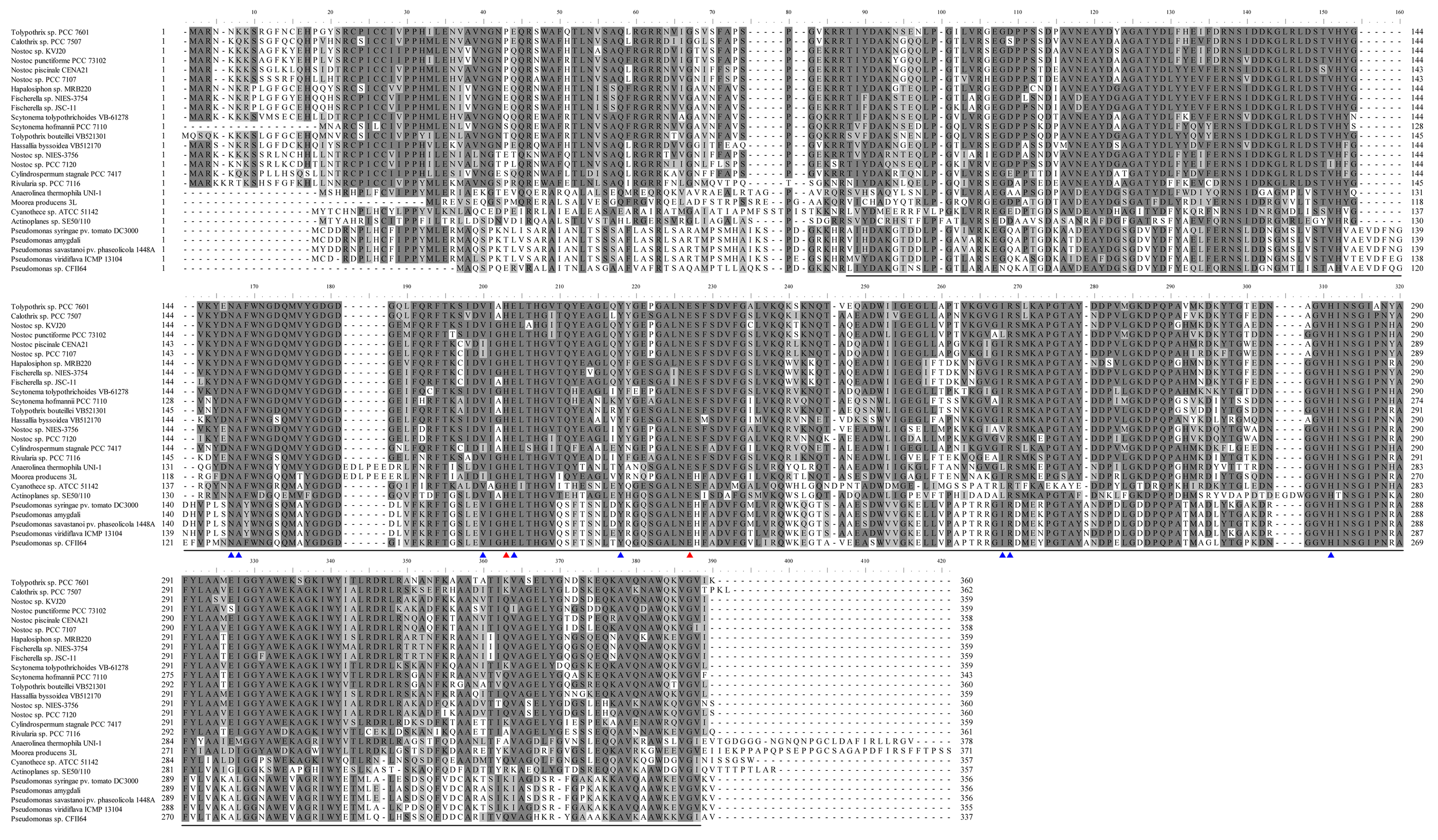

Supplement: S8 Fig — Red arrowheads are predicted zinc binding sites. Blue arrowheads are predicted active sites. Conserved M4 neutral protease domain is marked with black lines. (TIF) [file ppat.1007673.s011.tif]

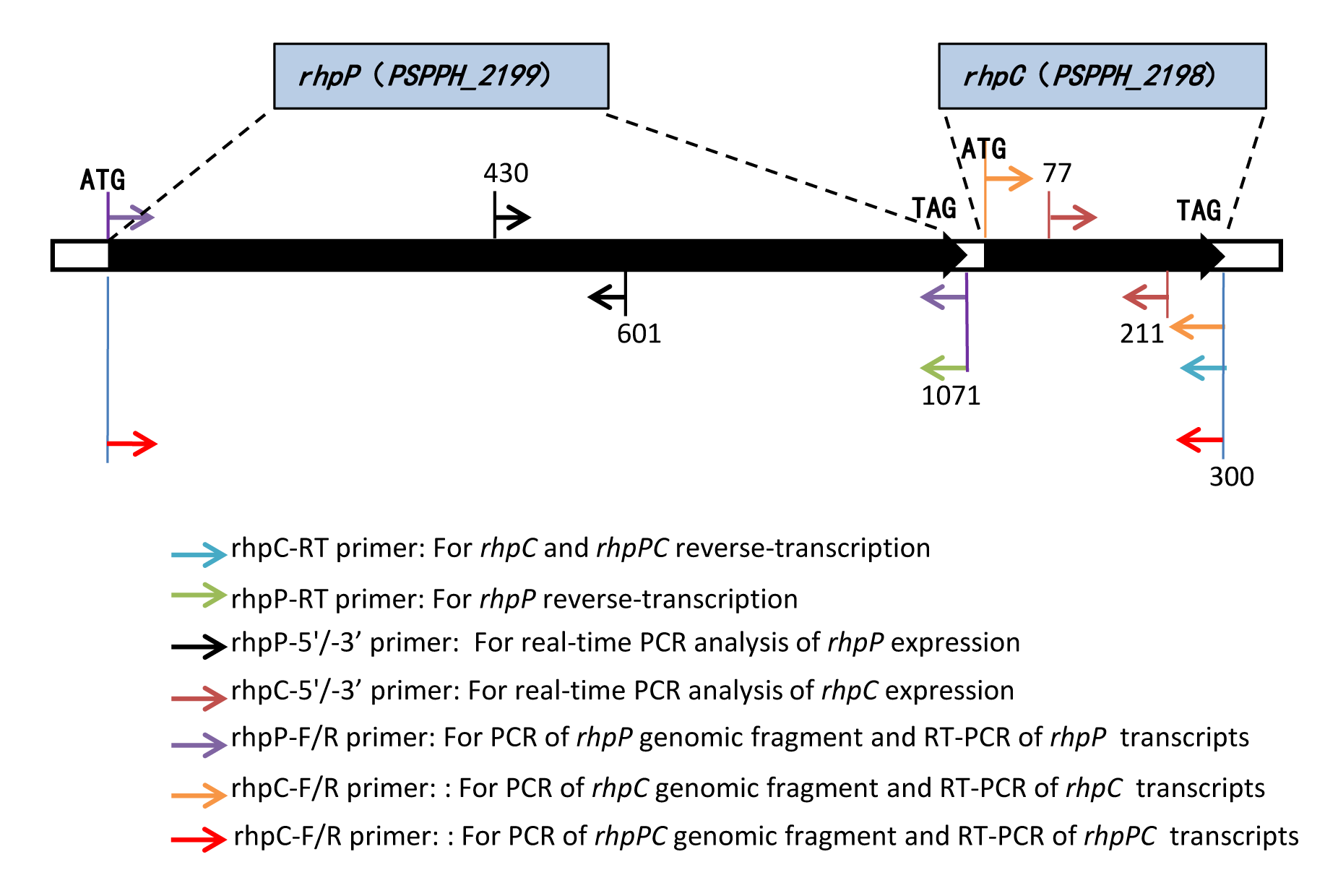

Supplement: S9 Fig — Primer pairs for different PCR analyses are coded with different colors. The sequences for the PCR primers are listed in Table S1. (TIF) [file ppat.1007673.s012.tif]

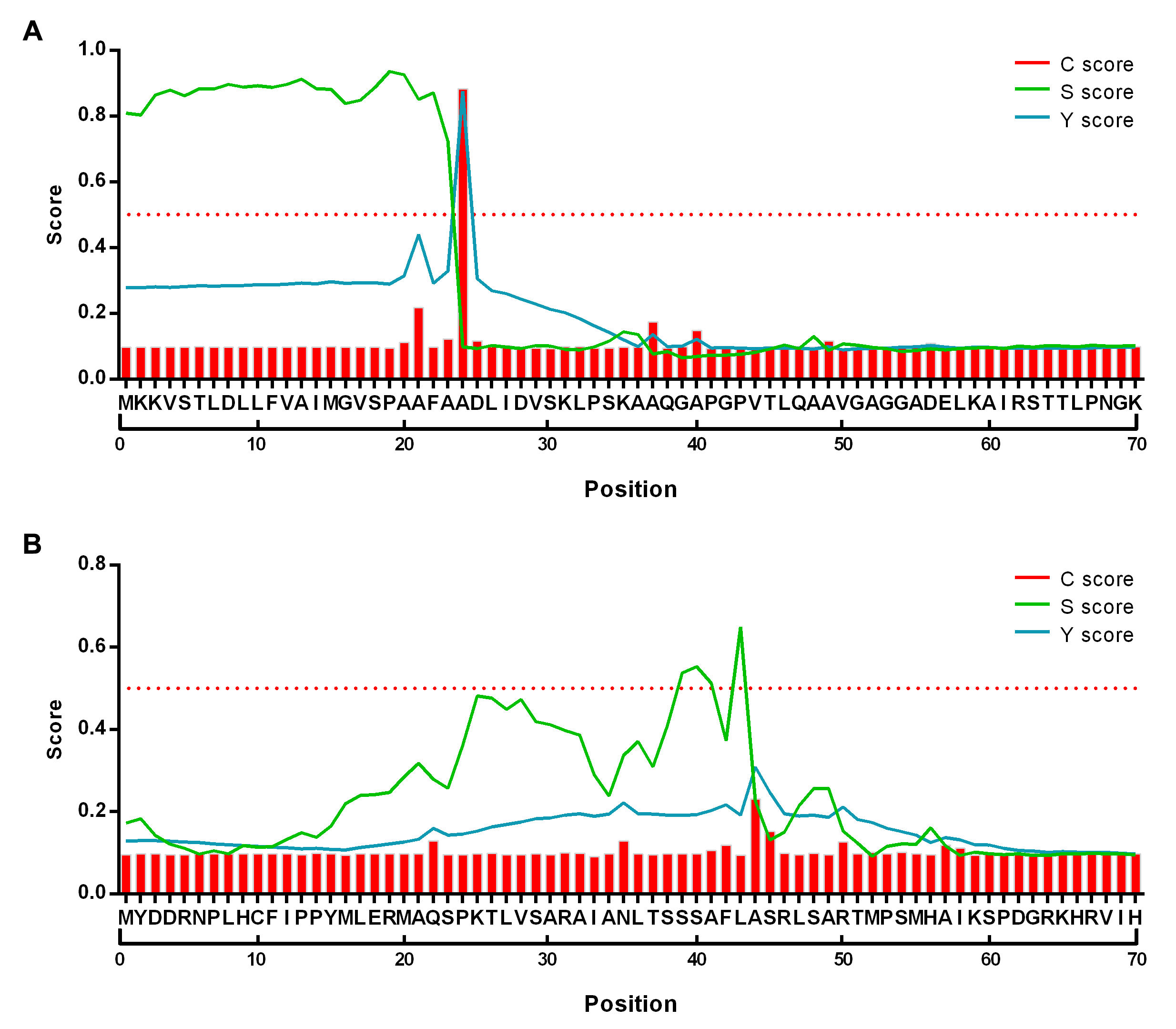

Supplement: S11 Fig — (A) Elastase of Pseudomonas aeruginosa to show predication of a typical signal peptide. (B) SignalP prediction of RhpP from Psph. (TIF) [file ppat.1007673.s014.tif]

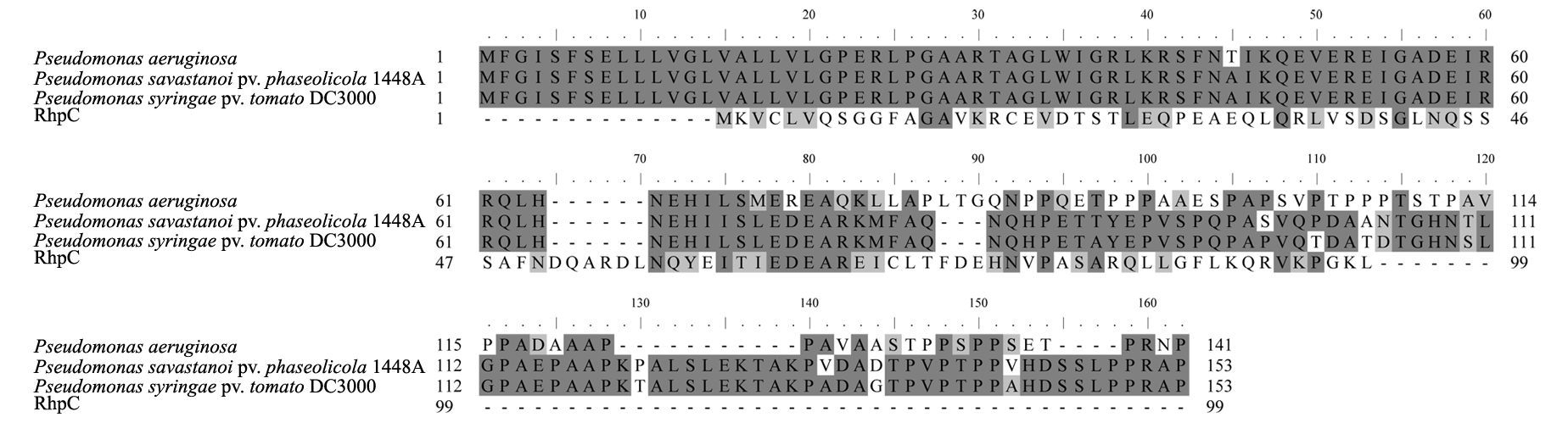

Supplement: S13 Fig — (TIF) [file ppat.1007673.s016.tif]
